# The value of what’s to come: Neural mechanisms coupling prediction error and the utility of anticipation

**DOI:** 10.1126/sciadv.aba3828

**Published:** 2020-06-19

**Authors:** Kiyohito Iigaya, Tobias U. Hauser, Zeb Kurth-Nelson, John P. O’Doherty, Peter Dayan, Raymond J. Dolan

**Affiliations:** 1 Max-Planck UCL Centre for Computational Psychiatry and Ageing Research, 10-12 Russell Square, London WC1B 5EH, UK.; 2 Gatsby Computational Neuroscience Unit, University College London, 25 Howland Street, London W1T 4JG, UK.; 3 Division of Humanities and Social Sciences, California Institute of Technology, 1200 E California Blvd, Pasadena, CA 91125, USA.; 4 Wellcome Centre for Human Neuroimaging, University College London, 12 Queen Square, London WC1N 3BG, UK.; 5 Deepmind, 6 Pancras Square, London N1C 4AG, UK.; 6 Max Planck Institute for Biological Cybernetics, 72076 Tubingen, Germany.; 7 University of Tübingen, 72074 Tübingen, Germany.

## Abstract

Having something to look forward to is a keystone of well-being. Anticipation of future reward, such as an upcoming vacation, can often be more gratifying than the experience itself. Theories suggest the utility of anticipation underpins various behaviors, ranging from beneficial information-seeking to harmful addiction. However, how neural systems compute anticipatory utility remains unclear. We analyzed the brain activity of human participants as they performed a task involving choosing whether to receive information predictive of future pleasant outcomes. Using a computational model, we show three brain regions orchestrate anticipatory utility. Specifically, ventromedial prefrontal cortex tracks the value of anticipatory utility, dopaminergic midbrain correlates with information that enhances anticipation, while sustained hippocampal activity mediates a functional coupling between these regions. Our findings suggest a previously unidentified neural underpinning for anticipation’s influence over decision-making and unify a range of phenomena associated with risk and time-delay preference.

## Teaser

Hippocampal-midbrain circuit boosts the utility of anticipation in the prefrontal cortex and drives information-seeking behavior.

## INTRODUCTION

“*Pleasure not known beforehand is half-wasted; to anticipate it is to double it*.”

– Thomas Hardy, *The Return of the Native*

Standard economic theory suggests that a reward is more attractive when it is imminent (e.g., eating now) than when it is delayed (e.g., eating tomorrow), predicting that people will always consume a reward immediately. This so-called temporal discounting has been adapted with great success, for instance, in the design of artificial intelligence systems that can plan their future effectively through to understanding aspects of the human mind.

However, real-life behavior is more complex (*1*–*3*). Humans and other animals will sometimes prefer to deliberately postpone a pleasant experience [e.g., saving a piece of cake for tomorrow or delaying a one-time opportunity to kiss a celebrity (*1*)], contradicting predictions of simple temporal discounting.

An influential alternative idea in behavioral economics is that people enjoy, or savor, the moments leading up to reward (*1*, *2*, *4*–*7*). That is, people experience a positive utility, referred to as the utility of anticipation, which endows with value the time spent waiting for a reward. Anticipatory utility is different from the well-studied expected value of the future reward (i.e., a discounted value of the reward) in standard decision and reinforcement learning theory, where the latter’s utility arises solely from reward and not from its anticipation. Crucially, in the theory of anticipatory utility (*1*), the two separate utilities (i.e., anticipation and reward) are added together to construct the total value. The added value of anticipatory utility naturally explains why people occasionally prefer to delay reward (e.g., because we can enjoy the anticipation of eating a cake until tomorrow by saving it now) (*1*), as well as a host of other human behaviors such as information-seeking and addiction (*4*, *8*).

Despite the theory’s clear mathematical formulation and its explanatory power for behavior, we know little about how the utility of anticipation arises in the brain. Although previous studies have described neural activity in relation to the expectation of future reward (*5*, *9*–*14*), it is not clear if or how such activity relates to the utility of anticipation. One major reason for this knowledge vacuum is the challenge in establishing behavior that is driven by the utility of anticipation in a laboratory setting [please also see (*5*)]. Notably, recent studies (*6*–*8*) have established a strong link between the utility of anticipation and information-seeking behavior, and this now has allowed us to formally test how the brain dynamically constructs anticipatory utility.

Here, we investigated the neurobiological underpinnings of value computation arising from the utility of reward anticipation and how acquired information modulates this anticipatory utility. In doing so, we combine a behavioral task, computational modeling, and functional magnetic resonance imaging (fMRI). We fit our computational model (*8*) of anticipation utility (*1*) to task behavior, and for each participant used the best model to make predictions about the time course of anticipatory utility in the brain. We then compared this predicted signal with actual fMRI data, finding that the ventromedial prefrontal cortex (vmPFC) encoded the temporal dynamics of an anticipatory utility signal, while dopaminergic midbrain encoded a signal reporting changes in reward expectation. This reward prediction error (RPE) is widely interpreted as a teaching signal in reinforcement learning theory (*15*), but our model predicts that it can act also to enhance an anticipatory utility, which, in turn, drives behavior. We show that hippocampus mediates this enhancement of utility and is a substrate for a functional coupling between the vmPFC and the dopaminergic midbrain (*16*, *17*). We suggest that these regions link reward information to the utility of anticipation, while a strong conceptual tie between the hippocampus, memory, and future imagination supports a suggestion from behavioral economics that the utility of anticipation relates to a vivid imagination of future reward (*18*–*20*).

## RESULTS

### Participants prefer to receive advanced information about upcoming reward

**Fig. 1:**
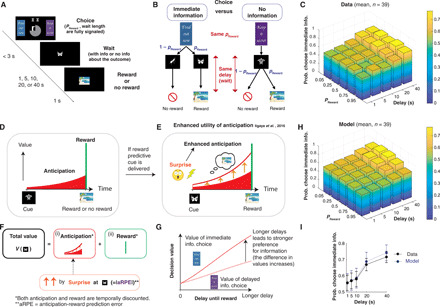
The utility of anticipation drives a preference for advanced information. (**A**) Task. Participants were presented with an immediate-information target (“Find out now”) and a no-information target (“Keep it secret”), as well as two central stimuli signaling the probability of reward and the duration of a waiting period until reward or no-reward delivery. A symbolic image cue was presented for the entire waiting period until a rewarding image or an image signaling no reward appeared. (**B**) The immediate-information target was followed by cues that predict upcoming reward or no reward (reward predictive cue or no-reward predictive cue). The no-information target was followed by a cue that implied nothing about the reward outcome (no-information cue). (**C**) Average behavior. Participants showed a stronger preference for advanced information under longer delay conditions [two-way analysis of variance (ANOVA), *F*_4,950_ = 10.0]. The effect of reward probability (*F*_4,950_ = 0.35, *P* > 0.05) showed heterogeneous dependencies (fig. S4). (**D** to **G**) Computational model (*8*). (D) Following (*1*), the value of each cue is determined by the sum of (i) the utility of anticipation that can be consumed while waiting for reward (red) and (ii) the value of reward consumption itself (green). (E and F) If a reward predictive cue is presented, then the anticipation is boosted throughout the delay period (orange upward arrows). The boosting is quantified by surprise, proportional to the absolute value of aRPE Eq. 1. (G) The model predicts that the value difference between the two targets is larger under longer delay conditions (*8*). (**H**) The average of modeled preferences, using a hierarchical Bayesian fitting procedure (*8*). (**I**) The model (blue) captures the effect of delay conditions in data (black). The error bars indicate the mean and SEs of participants (*n* = 39). See fig. S2 for the effect of probability conditions, and fig. S1 for how other classical models fail to explain behavior.

We used a variant of the behavioral task that has previously been linked to the utility of anticipation (Fig. 1A). In brief, our task examines how participants change their preference for resolving uncertainty about future pleasurable outcomes, based on reward probability and delay duration until an outcome (please also see Materials and Methods). Participants made decisions with full knowledge regarding conditions (probability, and delay, of reward outcomes), which were signaled with simple visual stimuli on each trial. The conditions were randomly selected for each trial—the probability was sampled uniformly at random from 0.05, 0.25, 0.5, 0.75, and 0.95, and the duration of a waiting period until reward or no-reward delivery was sampled uniformly at random from 1, 5, 10, 20, and 40 s.

On each trial, participants chose between an immediate-information target (labeled “Find out now”) and a no-information target (“Keep it secret”). If the immediate-information target was chosen, one of two cues, each of which uniquely signaled if reward would or would not arrive, was shown during the waiting period (Fig. 1B, left). If the no-information target was chosen, then a separate nonpredictive cue that carries no information about an upcoming outcome was shown on the screen during the waiting period (Fig. 1B, right), eventually followed by either reward or no reward. The reward image was randomly drawn from previously validated rewarding pictures (*8*, *21*) and consequently subject to immediate consumption (by viewing) upon delivery. The no-reward outcome was signaled by a neutral image.

In this design, participants’ choices did not affect either the final reward outcome or the duration of delay (Fig. 1B). Both reward probability and delay duration were predetermined and signaled to participants at the beginning of each trial. Participants could only choose if they want to gain knowledge about whether they would receive a reward or not before a delay. Therefore, standard decision theories that aim to maximize the chance of receiving rewards would predict no preference over these two choices, because the probability of obtaining a reward (hence, the expected value) is the same across the two choices (please see fig. S1, A to C). Thus, models with conventional temporal discounting predict no choice preference.

Contrary to the predictions of conventional theory, we found that participants exhibited a preference for advanced information. Further, consistent with previous findings (*22*–*24*), the preference for immediate information increased with the duration of a delay (Fig. 1, C and I) (*8*, *25*).

### Our computational model that links advanced information to the utility of anticipation accounts for behavioral data

Previous studies (*6*, *8*) have shown that the preference for obtaining advanced information can be accounted for by an economic notion of the utility of anticipation (*1*, *2*, *4*, *5*, *26*). While standard value-based decision theories assign values to the consumption of the reward itself, theories of the utility of anticipation also assign utility values to the moments that lead up to the receipt of reward (Fig. 1D; see Eq. 1 in Materials and Methods). One possible psychological root for the utility arising from reward anticipation is the pleasant subjective feeling while waiting for pleasant outcomes (*1*), although the mathematical framework of the anticipatory utility is open to wider interpretations [e.g., see (*4*)]. Here, our goal was to fill a current gap in our understanding by identifying neural processes that mediate a utility of anticipation.

Although the utility of anticipation naturally accounts for why people will delay the receipt of a reward (because they can consume anticipatory utility while waiting), the original formulation does not necessarily explain a preference for obtaining advanced information regarding a probabilistic outcome. The model still predicts indifference between the two choices in the task, because the utility of anticipation is linearly scaled with the probability of reward (as is the case for the expected value of the actual outcome), leading to the same average values for two choices (*8*) (illustrated in fig. S1, D to F). This is expected because information plays little role in the original formulation.

To better account for anticipatory utility, we recently proposed, and validated, a slight modification to this original formulation (*8*). Consider a case in which a future reward may or may not be delivered, but an early signal resolves the uncertainty, telling participants that a reward will be provided with certainty. The modification to the theory is that the utility of anticipation of a future reward is enhanced by the (in this case, positive) prediction error associated with the information signal. This surprise-based enhancement of anticipatory utility is inspired by experimental observations that such unexpected information can lead animals to become excited and will remain so until a reward arrives (*25*). Animals waiting for a certain reward with no such information do not show a similar level of excitement (*25*). The outcome of this can entail animals paradoxically preferring a less rewarding (on average), but more surprising, choice [e.g., (*3*, *25*)].

We mathematically formulated the surprise that relates to the enhancement of anticipatory utility by using a notion of RPE. Every time participants received advanced information about future reward (or no reward), participants experienced an RPE, defined by the difference between (i) the value of future that is just updated on the basis of the arrival of new information and (ii) the value of future that was expected before the arrival of new information. In standard theory, RPE is computed from the value of reward; in our model, it is computed from the utility of anticipation and reward (Eq. 5). Therefore, we refer to our model’s prediction error signal as an anticipation + reward prediction error (aRPE) signal. In our computational model, this aRPE quantifies a surprise that links to an enhancement (boosting) of anticipatory utility. Following the conventional mapping of prediction error to surprise (*27*), the model quantifies surprise by the absolute value of the aRPE, because unexpected negative outcomes (negative aRPE) can be just as surprising as unexpected positive outcomes (positive aRPE). This also avoids unreasonable effects such as turning negative anticipation to positive anticipation by multiplying with a negative aRPE. Thus, one of the simplest expressions for boosting is to assume that anticipatory utility is linearly enhanced by the absolute value of aRPE (please see Eqs. 1 and 2 in Materials and Methods).

It is important to note that an aRPE (or a standard RPE) is expected to be a phasic signal that lasts only for a short period. However, animals can remain excited throughout a whole anticipatory period (*25*), and so in the model, the enhancement of anticipation is sustained throughout a waiting period (*8*) (Eqs. 1 and 2). Therefore, the model predicts that a signal that is associated with boosting anticipatory utility will be a prolonged representation of the absolute value of aRPE (or a prolonged signal that is proportional to the amount of surprise). Such a signal is likely to be encoded in regions other than those encoding phasic aRPEs. We return to this question later.

In our task, the cue predictive of a future outcome that follows the immediate-information target creates a dopaminergic aRPE, and it triggers a boosting of the utility of anticipation. On the other hand, the nonpredictive cue following the no-information target does not generate aRPE and consequently does not trigger any boosting (fig. S1, G to I). Therefore, the model predicts that participants experience enhanced anticipatory utility after receiving a reward predictive cue following the immediate-information target, while they experience a default amount of anticipatory utility weighted by the probability of reward after receiving a no-information cue following the no-information target. Because of the sustained boosting, the model predicts that the difference in the values between the immediate-information target and the no-information target is larger under longer delay conditions (at least in the absence of strong discounting), causing an enhanced preference for the immediate information target at longer delay conditions (Fig. 1G) (*8*).

We fit this model to participants’ trial-by-trial behavioral data using a hierarchical Bayesian scheme (*8*) (see Materials and Methods). This method estimates group-level distribution over all participants, allowing us to have reliable estimates of each individual’s parameters without overfitting and to make fair model comparisons using sampling. As before (*8*), the model captured participants’ preferences for advanced information (Fig. 1, H and I). In particular, the model quantitatively captured the key feature of the data, which is an increase in preference for immediate information under longer delay conditions (Fig. 1I), as well as the preference over probability conditions (fig. S2). We also found that in addition to positive value to reward, participants assigned a negative value to the no-reward outcome, which creates a negative anticipatory utility associated with the no outcome (*8*). This allows the model to avoid advanced information if a participant assigns a large negative value [please see (*8*) for further evidence].

Other standard models do not capture this preference for advanced information. For example, models with discounted reward but with no anticipatory utility, or models with both discounted reward and anticipation utility but no enhancement of anticipation, cannot capture the observed behavior (please see fig. S1 for illustration). We formally tested this by fitting other possible models to the behavioral data using a hierarchical Bayesian method and compared the models’ integrated Bayesian information criterion (iBIC) scores through sampling from group-level distributions (*8*) (please see Materials and Methods). These analyses strongly favored our full model over other standard computational models (fig. S3).

In addition to the task behavior outlined here, our model also captures a wide range of existing findings related to information-seeking behavior (*3*, *6*, *25*, *28*), with potential links to addiction and gambling (*8*) (also see Discussion). However, an impressively rich and sophisticated literature describing neural correlates for an expectation of future reward (*5*, *9*–*14*) has, with only a few notable exceptions [see (*5*)], focused mainly on standard issues of temporal discounting. Consequently, this literature does not address a separate and additional boosted anticipatory utility term (see Materials and Methods for details) that, as described above, is necessary to explain a wide range of reward-related behavior.

Therefore, we next sought to elucidate the neurobiological basis of value arising from anticipation, using our computational model that captures participants’ behavior. In particular, three key components of our model were of interest: the representation of anticipatory utility during waiting periods, the aRPE signal at advanced information cue presentation, and a sustained boosting signal of anticipation during waiting periods following surprise. To identify a unique signal for anticipatory utility, we regressed out other related signals, such as the expected value of a future reward. Last, we examined how brain regions encoding these computational components are coupled together to dynamically orchestrate the utility of anticipation, including how the brain links the arrival of reward information to the utility of anticipation.

It is important to note that our computational model is a general mathematical formulation that does not specify the psychological roots of anticipatory utility. This is analogous to standard reinforcement learning models encompassing very complex psychological roots of reward value (*29*). Our goal was to elucidate the neural correlates of our computational model’s mathematical predictions about how advanced information links to the values arising during anticipatory periods, which, in turn, drive behavior. We discuss the possible psychological roots of anticipatory utility in Discussion.

### The vmPFC encodes the utility of anticipation

**Fig. 2:**
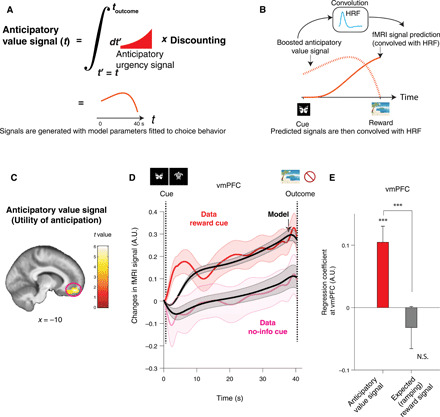
Neural representation of our computational model’s anticipatory utility signal in the vmPFC. (**A**) The anticipatory utility signal at time *t* is an integral of discounted future anticipation (urgency signal) at *t*′ > *t* (red curve). This signal is different from a well-studied expected value of future reward, which we included in the same GLM. (**B**) The model’s prediction for fMRI signals (solid red) is computed by convolving the model’s signal (dotted red) with a canonical HRF (light blue). (**C**) BOLD in vmPFC positively correlated with an anticipatory utility signal. This survived our phase-randomization test (whole-brain FWE *P* < 0.001; see fig. S8) and SPM’s standard whole-brain FWE (*P* < 0.05). A cluster surrounding the peak [10,50,16] (cFWE, *P* < 0.05 with height threshold at *P* < 0.001) is shown for display purposes. (**D**) The temporal dynamics of the BOLD signal in the vmPFC [shown in (C)] matched the model’s anticipatory utility signal during the anticipation period. Changes in activity following receipt of a reward predictive cue (red) and a no-information cue (magenta), as well as the model’s prediction for each of these conditions (black) are shown. The error bar indicates the SEM over participants. (**E**) A confirmatory analysis shows that activity in vmPFC is more strongly correlated with our model’s anticipatory utility signal than an expected reward value signal. The average regression weights in the vmPFC for the anticipatory utility signal were significantly greater than the expected reward signal (***P < 0.001, permutation test). The former was also significantly larger than zero (***P < 0.001, *t* test, *t*_38_ = 4.07), but the latter was not. The error bars indicate the mean and SEM. A.U., arbitrary units; N.S., not significant.

Our model predicts that the signal of anticipatory signal dynamically changes throughout a delay period (Eq. 11 in Materials and Methods). Regardless of boosting, the signal ramps up as the outcome approaches, but the value is also subject to conventional discounting. This implies a tilted inverted-U shape over time under typical parameter settings (Fig. 2A).

On the basis of our hierarchical model fit to choice behavior, we calculated each participant’s maximum a posteriori (MAP) parameters within the computational model. Using these parameters, we estimated subject-specific time courses of several variables that we tested on neural data. The predictions include (i) anticipatory utility value during waiting periods (Eq. 11 in Materials and Methods), (ii) discounted outcome value (standard expected value) during the same waiting periods (Eq. 13 in Materials and Methods), and (iii) prediction errors at cue presentation (Eq. 17 in Materials and Methods). These signals were convolved with SPM’s (statistical parametric mapping) default canonical HRF (hemodynamic response function) (Fig. 2B; see fig. S5 for an example). As illustrated in Materials and Methods, we separated predictive anticipatory signals for positive reward and no reward, because we found that participants assigned a negative value to no-reward outcome (*8*). SPM’s directional orthogonalization for parametric regressors was turned off throughout data analysis here.

Note that previous studies into value computation (including of temporal difference learning) have focused on the current value of the expected future reward. This quantity is usually closely correlated with the quantity that is the focus of our current study, namely, the additional anticipatory utility associated with future reward (fig. S5). Thus, a brain signal correlated with the anticipatory utility might conventionally be classified as a correlate of the expected value of a future reward. Here, by including these regressors together in the same general linear model (GLM), we could identify unique correlates for the utility of anticipation. We excluded trials with a short waiting time (1 s) from the analysis to separate effects of responses to cues.

We found that the model’s anticipatory utility signal for positive reward correlated significantly with blood oxygen-level dependent signal (BOLD) in vmPFC {*P* < 0.05, whole-brain familywise error (FWE) correction; peak Montreal Neurological Institute (MNI) coordinates [10,50,16], *t* = 6.02; Fig. 2C} and in caudate (*P* < 0.05, whole-brain FWE correction; peak coordinates [−20, −2,18], *t* = 5.81; fig. S6). These results are consistent with a representation of the value of imagined reward reported previously in vmPFC (*30*, *31*) and of reported anticipatory activity in vmPFC (*5*, *13*, *32*) and in caudate (*9*). Across the brain, we found no significant effect of anticipatory utility arising from no-reward outcome that survived a stringent whole-brain correction (see fig. S7). Thus, we focus on the anticipatory utility of future reward referred to henceforth as anticipation utility.

Given the importance of avoiding potential false positives from autocorrelations in slowly changing signals (*33*), we conducted nonparametric, phase-randomization tests where we scrambled the phases of signals in a Fourier decomposition (fig. S8A) (*34*, *35*). This test can be applied to neuroimaging and electrophysiology studies, so as to avoid false-positive discoveries, particularly when analyzing correlations between slow signals such as values (*33*, *35*). To do so, we transformed our model’s predicted anticipatory utility signal for each participant into Fourier space, randomized the phase of each frequency component, and transformed the signal back to the original space. Only the regressor being tested was randomized, while others were kept the same in the full GLM. We then performed a standard analysis on this full GLM for each participant with the scrambled signal and then conducted a second-level analysis. By repeating this procedure many times, we created a null distribution. To protect this test against family-wise error, we constructed the null distribution by taking a maximum value of correlation score across a region of interest (ROI), or across the whole brain, from each of our second-level analyses, comparing against the correlation value in the original analysis. We found that the effects in vmPFC (*P* < 0.001, randomization whole-brain FWE-corrected) and caudate (*P* < 0.01, randomization whole-brain FWE-corrected) survived this Fourier phase-randomization test (fig. S8B; please also see fig. S9).

A more detailed inspection of these signals, during the waiting period, showed that the time course of vmPFC activity closely resembled our model’s predictions. In Fig. 2D, we plot the time course of average fMRI signals in the vmPFC cluster shown in Fig. 2C during the waiting period separately for two conditions, namely, when participants received a reward predictive cue (red) and when participants received a no-information cue (magenta). The time courses track the model’s predictions in each condition (black).

We note that a standard expected value of future reward signal was also included in the same GLM so that we can evaluate unique correlations for the utility of anticipation. Both signals showed similar ramping toward reward (please see fig. S5 for an example participant); therefore, anticipatory utility signals may have previously been classified as the expected value of future reward signal. As a confirmatory analysis, we compared the correlation of the vmPFC with our model’s anticipatory utility signal and to that with a standard expected reward signal. In Fig. 2E, we plotted average β values in the vmPFC cluster for the anticipatory utility and for the standard expected reward (note that both regressors are present in the same GLM) and confirmed that the difference between the coefficients was significant (*P* < 0.001, permutation test). We stress that this is a confirmatory analysis, because we already know that vmPFC is significantly correlated with the anticipatory utility and not with the expected value signal. The model’s expected reward signal was instead correlated significantly with regions, including the superior temporal gyrus (*P* < 0.05, whole-brain FWE correction; [−48, −48,16], *t* = 5.28; fig. S10A). This also survived a phase-randomization test (*P* < 0.001).

We also tested whether our found signal is distinct from a more generic ramping signal, such as a linear ramping signal. To test this, we added a regressor that ramps up linearly in each anticipatory period to the original GLM and compared the average β coefficients of this regressor against that of the utility of anticipation. We confirmed that the coefficients of the utility of anticipation are significantly larger than those of the linear ramping signal (fig. S11), supporting that our results show neural correlates of the utility of anticipation, instead of other types of ramping signals.

We further asked whether BOLD in the vmPFC during the waiting period correlated with a simpler signal, such as constant expected outcome value. When the immediate-information cue is presented, this is the same as the value of reward or no reward without discounting or anticipatory modulation; otherwise, it is an average of the values of reward and no reward weighted by their respective probabilities. We examined the singular contribution of this signal by adding it as another parametric boxcar regressor during waiting periods to the original GLM and then comparing the average β values of the vmPFC cluster between the anticipation utility and the expected value, regressor. In this way, we estimated the partial correlation of each regressor. As shown in fig. S12, vmPFC BOLD was more strongly correlated with the model’s anticipatory utility signal than with the constant expected value signal (*P* < 0.001, permutation test). BOLD was still positively correlated with the model’s anticipatory utility signal (*P* < 0.001, *t* test, *t*_38_ = 3.93), and the effect of an expected value signal was not significant. We again note that this is a confirmatory analysis.

For completeness, we report descriptively that an anticipatory urgency signal, which is an anticipation signal before integration (Eq. 15 in Materials and Methods), correlated with anterior insular cortex (*11*) ([34,30,2], phase-randomization test, *P* < 0.01; fig. S10B).

### The dopaminergic midbrain encodes our computational model’s aRPEs at the time of advanced information cues

The aRPE arising at advanced information cues is a unique and critical signal in our model. First, unlike conventional models relying on reward, our model’s aRPE is computed from the value arising from both reward anticipation and reward itself (Eq. 5 in Materials and Methods). Second, while in a standard reinforcement learning model, an RPE serves as a learning signal; in our model, it triggers a surprise that is associated with enhancement (boosting) of anticipatory utility (Eq. 2 in Materials and Methods). In this regard, aRPE also differs from a conventional temporal difference prediction error signal (*15*), which considers conventionally discounted outcomes alone and does not involve boosting. Rather, our computational model’s aRPE signal encompasses both a standard RPE and the so-called information prediction error (IPE) (*23*, *24*, *36*, *37*), both of which have been shown to be represented in the activity of dopamine neurons (*23*). Dopamine has also been implicated in enhanced motivation [e.g., (*38*)]. Therefore, on the basis of extensive prior studies, we hypothesized that an aRPE signal arising at the time of advanced information cues would be encoded in the midbrain dopaminergic regions and ventral striatum [e.g., (*10*, *23*, *39*)].

For this, using each participant’s MAP parameter estimates obtained from fitting our model to choice behavior, we calculated a full, signed, aRPE signal, at the onset of advanced information cues (reward predictive, no-reward predictive, and no-information cues), based on the discounted utility of anticipation (including both positive and negative cases) and that of outcomes (Eq. 17).

We assumed that participants fully learned the task in the training period. Therefore, the size of aRPE was determined entirely by each trial’s experimental conditions (probability and delay of the reward) as well as the model’s fitted parameters, meaning that an aRPE was not affected by recent trials’ outcomes. Therefore, we analyzed the fully self-consistent aRPE (please see Materials and Methods, Eq. 17).

**Fig. 3:**
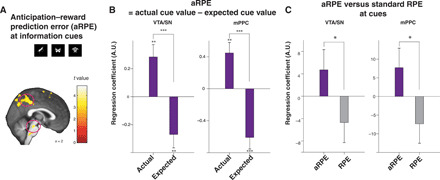
Neural representation of our computational model’s aRPE signals. (**A**) The ventral tegmental area and substantia nigra (VTA/SN) and medial posterior parietal cortex (mPPC) BOLD positively correlated with the model’s aRPE at the time of advanced information cue presentations [VTA/SN, *P* < 0.05 FWE small volume correction (*39*); mPPC, *P* < 0.05 whole-brain FWE, cluster-corrected at *P* < 0.001]. Voxels at *P* < 0.005 (uncorrected) are highlighted for display purposes. (**B**) Our confirmatory analysis shows that both the VTA/SN and the mPPC show paradigmatic correlations with aRPE. At the time of advanced information cue presentations, BOLD in the VTA/SN and the mPPC positively correlated with the model’s actual cue value signal and negatively with the model’s expected cue value signal, indicating that both regions express canonical prediction errors. The difference was significant in the VTA/SN (*P* < 0.001, permutation test) and in the mPPC (*P* < 0.001, permutation test). The positive correlation with cue outcome values and the negative correlation with expected values were all significant (received cue value, *P* < 0.01 for the VTA/SN and the mPPC by *t* test, *t*_38_ = 3.24 and *t*_38_ = 3.40; expected cue value, *P* < 0.01 for the VTA/SN and *P* < 0.001 for the mPPC by *t* test, *t*_38_ = 2.82 and *t*_38_ = 4.37). (**C**) Our confirmatory analysis shows that both regions express stronger correlations with our model’s full aRPE than with standard prediction error with discounted reward (RPE) at advanced information cues. The difference was significant between the VTA/SN and in the mPPC cluster (*P* < 0.05, permutation test). ***P < 0.001, **P < 0.01, and *P < 0.05.

We found that the model’s signal correlated significantly with BOLD in a midbrain dopaminergic region, encompassing the ventral tegmental area and substantia nigra (VTA/SN) [Fig. 3A; *P* < 0.05, small volume FWE correction with an anatomical ROI; (*39*) [4, −26, 20], *t* = 3.78]. We analyzed VTA/SN with an anatomical ROI following previous literature (*39*). We note that this correlation at VTA/SN also survives FWE correction over the extended ROI that covers two regions: VTA/SN and ventral striatum (*39*) (*P* < 0.05, FWE small volume correction). In addition, we also found that BOLD in the medial posterior parietal cortex (mPPC) (*40*) correlated significantly with the model’s predicted signal (Fig. 3A; *P* < 0.05, cluster-level whole-brain FWE correction with the height threshold *P* < 0.001; *k* = 166, peak at [0, −42, 50]). We did not find significant associations in ventral striatum, perhaps because cue and reward onsets were unusually temporally distant (up to 40 s), a finding consistent with a previous report that ventral striatum is not relevant for learning when feedback is delayed (although hippocampus is) (*41*). Further, we explored whether locus coeruleus (LC) is correlated with this signal; however, we did not find a significant effect.

Previous studies suggest that significant correlations reported between fMRI signals and prediction errors might be attributable to strong correlations with actual cue value alone, regardless of the presence of negative correlations with expected cue value (*42*). To rule out this possibility, we performed a confirmatory analysis by constructing a GLM with separate regressors for the model’s values of presented cue values and the model’s expected cue values, both of which were computed from the utility of anticipation and reward (Eq. 5). The average regression coefficients correlated positively with the model’s (actually presented) cue value and correlated negatively with the model’s expected (average) cue value (Fig. 3B in both the VTA/SN and in the mPPC clusters shown in Fig. 3A). Thus, responses in these regions had the characteristic of canonical prediction error signals (*42*).

Because our model’s aRPE signal, with the values of anticipation and reward, is more complex than a standard RPE signal with reward value alone, we performed a further confirmatory analysis. Here, we constructed a GLM that included the model’s full aRPE signal (Eq. 8) and a standard RPE error signal based exclusively on reward values (Eq. 19). We then compared the partial correlations associated with these regressors. We found in both VTA/SN (*39*) and the mPPC cluster that the average partial correlation is greater for our model’s full aRPE signal than for the standard RPE signal with discounted reward value alone (Fig. 3C).

Last, BOLD in the mPPC has previously been reported to covary with a simpler prediction error signal, the state prediction error (SPE) signal (*43*). In our experiment, this SPE signal is the absolute value of the difference between outcome (1 or 0) and expectation (the presented probability of reward; Eq. 18). To rule out SPE as a driver of our results, we performed a confirmatory analysis, by constructing a GLM that included the model’s full aRPE signal and its SPE signal and then compared the β values of partial correlations associated with these regressors. For both the VTA/SN (*39*) and the mPPC cluster, the average partial correlation weights for the model’s full RPE were greater than those for the SPE signal (fig. S13).

### The hippocampus correlates with our computational model’s surprise that can enhance the utility of anticipation

**Fig. 4:**
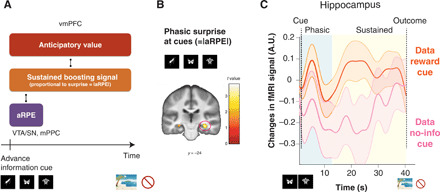
Neural correlates of our computational model’s surprise that can boost anticipation utility in our computational model. (**A**) Our model predicts that a surprise, quantified by the absolute value of aRPE, can boost the utility value of anticipation. The model predicts the effect of boosting to be sustained during the anticipatory period, in contrast to the phasic, short, aRPE signal. (**B**) A surprise at advanced information cues, quantified by the absolute value of aRPE, significantly correlated with BOLD in the hippocampus [FWE, *P* < 0.05, small volume correction (*46*)]. (**C**) The temporal dynamics of fMRI signal in the hippocampus. Changes in activity averaged over participants after receiving a reward predictive cue (orange), and after receiving a no-information cue (magenta), are shown. The phasic response confirmed in (B) is apparent in the early phase of the delay period (blue). Still, the coding of boosting-related value is sustained throughout the entire waiting period (blue and yellow), which is what our model predicted. The error bar indicates the SEM. Please also see fig. S14 for responses to a no-reward predictive cue.

Our computational model predicts an enhanced anticipation utility following a surprise that is coincident with advanced information cues. The magnitude of this enhancement is proportional to the surprise, which is defined simply by the absolute value (*27*) of aRPE (Eq. 2 in Materials and Methods). Our model also predicts that any boosting should be sustained over the entire duration of a waiting period (Fig. 4A), unlike the phasic (a)RPE signals that we just examined (*23*, *44*).

Previous research suggests that the hippocampus is an ideal substrate for this effect. First, in the context of recognition tasks, the hippocampus encodes surprise (mismatch, novelty) signals [e.g., (*17*)]. In addition, the hippocampus is associated with learning for an association between cues and delayed feedback. Further, extensive studies implicate a coupling of the hippocampus with the VTA/SN and with the PFC [e.g., (*16*, *17*, *20*, *45*)], the two regions that we show are linked most to our model’s computation. Also, although we do not specify the psychological roots of our computational model’s enhancement of anticipation utility, we note that in the original study of anticipatory utility, the magnitude of anticipation utility is suggested to relate to the strength of imagination for future reward (*1*). Many studies link hippocampal activity to the imagination of future prospects [e.g., (*18*)], where prefrontal-medial temporal interactions influence the effects of imagination on valuation (*19*), as well as support the mental construction of future events (*20*).

Therefore, we first examined the phasic response of the hippocampus to a surprise at the onset of the advanced information cue presentation, quantified by the absolute value of the model’s aRPE. As predicted, we found that hippocampal activity was significantly correlated with the magnitude of a surprise {*P* < 0.05, FWE small volume correction by an anatomical mask of hippocampus; [32, −24, −12], *t* = 3.60; Fig. 4B (*46*)}. The phasic response to surprise is an important feature for the model’s boosting anticipation utility, but as outlined, the model predicts that activity associated with boosting should be sustained until ultimate reward delivery (Fig. 4A). We found that hippocampal activity in the cluster that responded phasically to surprise at cue (the cluster is taken at *P* < 0.05, FWE small volume correction from the analysis in Fig. 4B) was greater throughout the waiting period after a reward predictive cue was presented (in which case, a surprise was induced), compared to that following presentation of a no-information cue (in which case no surprise was induced), as seen in Fig. 4C (see also fig. S14 for responses to a no-reward predictive cue). This was quantified in fig. S15 (*P* < 0.05, permutation test). Thus, in addition to expressing the magnitude of a surprise at advanced information cues, hippocampal BOLD during the wait suggests features associated with our model’s signal that relates to boosting anticipation utility.

We also explored the possibility that amygdala correlates with the surprise at the cues. However, we found no voxel in amygdala showing significant correlations with this.

### The midbrain-hippocampus-vmPFC circuit implicates our model’s predicted coupling of prediction error (at advanced information) to the utility of anticipation

So far, we have shown that distinct regions encode our model’s computational signals. The vmPFC encodes our model’s utility value of anticipation; the VTA/SN (as well as the mPPC) encodes an aRPE signal that is associated with a trigger for boosting of the utility of anticipation, and the hippocampus encodes a sustained signal associated with our model’s boosting of the utility of anticipation. In our computational model, these three signals are functionally coupled (please see Figs. 1, E and F, and 4A for schematic illustrations and Eqs. 1 and 2 in Materials and Methods for a more precise mathematical description). Specifically, as illustrated in Fig. 4A, our model expects that a region that encodes a signal associated with a sustained effect of boosting should be functionally coupled both to a region encoding aRPE and to a region encoding the utility of anticipation. The hippocampal BOLD signal in Fig. 4C suggests that it encodes both phasic (related to aRPE) and sustained (related to anticipation utility) signals (fig. S15). Furthermore, extensive studies implicate functional couplings of hippocampus with the VTA/SN as well as with the PFC (*16*, *17*, *45*).

We hypothesized that sustained hippocampal activity mediates our model’s anticipation utility computation. In essence, to boost anticipation utility, the hippocampus links computations in the VTA/SN (aRPE) and the vmPFC (anticipation utility). If the hippocampus is coupled to both the VTA/SN and the vmPFC, then it should correlate with mixed variables (interaction) from the VTA/SN and the vmPFC. To formally test this idea, we analyzed functional connectivity using dual psychophysiological interaction (PPI) regressors based on two a priori seed regions: (i) the vmPFC (which encodes anticipation utility) and the model’s aRPE signal at advanced information cues (which is encoded at the VTA/SN) as a psychological variable, and (ii) the VTA/SN (which encodes aRPE) as a seed and the model’s anticipation utility signal (which is encoded at the vmPFC) as a psychological variable. The PPI was constructed in this manner because we wanted to test whether the hippocampus couples to both the VTA/SN and the vmPFC. Each of these two PPI regressors includes variables relating to both the vmPFC (anticipation) and the VTA/SN (aRPE), and these variables are coupled in our computational model through the notion of boosting; therefore, each regressor tests our hypothesis that the hippocampus links the VTA/SN (aRPE) and the vmPFC (anticipation) as a potential substrate of boosting. Thus, we included these two sets of regressors into the single GLM we used so far (see Materials and Methods) and tested whether hippocampal activity significantly correlated with these PPI regressors. We also explored the possibility that amygdala contributes to this interactive computation. However, we found no voxel in amygdala, showing significant correlations with either of the PPI regressors.

**Fig. 5:**
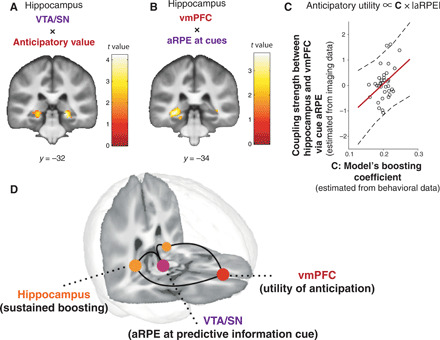
Functional connectivity analysis suggestive of a neural network associated with our model’s anticipation utility computation. (**A**) Functional coupling between the VTA/SN and the hippocampus is positively modulated by the model’s anticipation utility signal [*P* < 0.05, FWE small volume correction (*46*)]. PPI regressor: BOLD signal in VTA/SN modulated by model’s anticipation utility signal. (**B**) Functional coupling between the vmPFC and the hippocampus is positively modulated by the model’s aRPE signal [*P* < 0.05 FWE small volume correction (*46*)]. PPI regressor: BOLD signal in vmPFC modulated by the model’s aRPE signal. (**C**) The functional coupling strength between the vmPFC and the hippocampus mediated by the model’s prediction error signal is positively correlated with the model’s boosting coefficient parameter estimated by the behavior of participants (*r* = 0.37, *P* < 0.05). (**D**) Three distinctive regions contributed to construct the anticipation utility, in a manner that is predicted by our computational model. The three-dimensional brain image was constructed by the mean T1 brain images, which were cut at *y* = 34 and *z* = 15.

We found significant correlations in the hippocampus for both PPI regressors. Thus, the functional coupling between the VTA/SN (the area encoding aRPE) and the hippocampus was significantly modulated by our model’s anticipation utility signal {*P* < 0.05, FWE small volume correction; [22, −32, −6], *t* = 3.89; Fig. 5A (*46*)}. In addition, the functional coupling between the vmPFC and the hippocampus (*47*) was significantly modulated by our model’s aRPE signal {*P* < 0.05, FWE small volume correction; [−30, −34, −6], *t* = 3.70; Fig. 5B (*46*)}. We also performed a conjunction analysis to see whether the two regions that are correlated with two PPI regressors overlapped. However, we found null results, suggesting that coupling to the VTA/SN and to the vmPFC may be mediated by different subregions in the hippocampus.

If the hippocampal-vmPFC coupling mediates our computational model’s boosting of anticipation, then the coupling strength that we estimated in our PPI analysis should relate to the model’s magnitude of boosting that we estimated from choice behavior. Our model predicts that the magnitude of boosting is linearly correlated with a parameter *C*, the linear boosting coefficient (Eq. 3, Fig. 5C), which we had already fit to each participant. Therefore, we tested whether the linear boosting coefficient (that we estimated from our behavioral model-fitting) and the hippocampal-vmPFC coupling strength (that we estimated from our fMRI PPI analysis) are correlated with each other. As seen in Fig. 5C, we found that these two variables estimated separately from imaging and behavioral data are positively correlated across participants. This provides further evidence supporting the idea that this three-region network is involved in our model’s anticipatory utility computation. We note that we *z*-scored aRPE so that the size of aRPE is not directly correlated with a preference of advanced information.

We also note a recent study suggesting cautious attitudes when interpreting between-subjects correlation using model-based neuroimaging analysis (*48*). Although our analysis involves an interaction term (a PPI regressor), which itself includes a BOLD sequence, here, we aimed to test the proportional coding, whether the magnitude of functional coupling is correlated with the model’s parameter. To ensure that our correlation is not trivial, following (*48*), we tested whether there is a correlation between the model’s parameter *C* and the variance of the PPI regressor. Unlike the example given previously (*48*), we found no significant correlation between these two variables (fig. S16).

These functional connectivity results support our hypothesis that the hippocampus plays a key coordinating role in our model’s computation, that is, potentially boosting the utility of anticipation and linking the vmPFC’s encoding of the utility of anticipation with the VTA/SN’s encoding of prediction errors at advanced information. The findings point to these regions functioning as a large-scale neural network for linking advanced information to the utility of anticipation (Fig. 5D), driving a preference for advanced information in our task.

## DISCUSSION

The utility of anticipation has long been recognized as a critical notion in behavioral economics and the cognitive sciences. While it has been linked to a wide range of human behavior that standard reward value–based decision theories struggle to account for (e.g., a preference for advanced information, risk-seeking, and addiction), the neural basis of the theory is unknown. It is a different notion from the standard expected value of future reward (and we duly controlled for this standard value throughout our analyses). Here, we took advantage of a new link between computational theory and behavior and applied this perspective to fMRI data to uncover how the utility of anticipation arises in the brain and how advanced information links to the utility of anticipation (please see fig. S17 for a visual summary of Discussion).

Crucially, we show a network for computing the utility of anticipation and liking of advanced information, consisting of three specific brain regions. First, we show that vmPFC represents the time course of an anticipation utility signal that evolved separately from a standard reward expectation signal during a waiting period. Second, dopaminergic midbrain regions, encompassing VTA/SN, encoded the model’s aRPE that signals changes in expected utility of anticipation and reward at advanced information cues. Third, the hippocampus, whose activity indexed our model’s surprise signal, was functionally coupled both to vmPFC and to the VTA/SN, in a sustained manner consistent with our model’s predicted boosting of anticipation utility. While the three-region functional coupling has been previously implicated in other settings (*16*, *17*, *45*), our study provides evidence for an explicit, mathematically defined, computational role. We suggest that its role in the context of our study is to link advanced information to a utility of anticipation that works as a reinforcement for behavior.

Our study provides insights into neural processes underlying human decision-making that standard decision theories struggle to explain. A case in point, in our current study, concerns a preference for early resolution of uncertainty (*4*), also known as information-seeking (*22*–*24*, *49*), or observing (*50*, *51*). Humans and other animals are willing to incur costs to find out their true fate, even if this knowledge does not change actual outcome. An alternative idea, as opposed to that of boosted anticipatory utility, is the notion that people derive value from information itself (*23*). However, this so-called intrinsic value of information cannot explain why a preference for advanced information is valence dependent (*24*), that it depends on the reward probability in a way that does not covary with information-theoretic surprise (*52*), and manifests a sensitivity to delay until reward (as we also demonstrated here) (*8*, *25*). All of these findings are a natural consequence of the coupling of information to the utility of anticipation (but not of information per se).

Consequently, our results account for previous neural findings of the intrinsic value of information. This so-called IPE signal is presumed to arise from the value of information (*22*–*24*, *36*, *37*) and has been reported in the same midbrain dopaminergic regions as standard RPEs (*22*, *23*), implying that the two signals might be strongly related. Our model accounts for IPEs as a side effect of anticipation-dependent aRPE. We found that an aRPE signal correlates positively with BOLD signal in dopaminergic midbrain regions (*22*, *23*) and in the mPPC (*40*). We found that clusters in these regions are more strongly correlated with our model’s aRPE signal than with a standard RPE signal with no utility of anticipation. This implicates that these regions encode our aRPE signal that unifies standard RPE signals and IPE signals.

More broadly, our results offer alternative accounts for addiction and the possibility of individually tailored psychiatric interventions (fig. S17). While initial phases of addiction (*53*) involve excessive dopamine release at the time of drug consumption (*54*), later phases involve intense craving. Our model implies that people boost anticipation utility when a likelihood of drug administration increases (e.g., when purchasing drugs). People may feel greater value from obtaining drugs (which can act as a kind of conditioned stimuli) than from administering them, because the former includes utilities associated with an anticipation of future administration. Our model predicts that people with certain parameter values (e.g., large boosting coefficients) could repeatedly overboost the value of anticipating drugs, resulting in excessive, pathological, drug-seeking (see Eq. 10). Although the learning process leading to pathological behavior may be very slow in a natural world, by fitting our model to participants performing the task used here, we can, in principle, link an individual’s tendency toward addiction with a unique cause of this disorder (e.g., excessive boosting or imbalance between anticipation and discounting). This, in turn, can suggest interventions tailored to individual patients, such as cognitive behavioral therapy focusing on controlling anxiety and craving (*55*), as well as possible dopaminergic antagonists to control boosting.

Our study is relevant also for unifying separate notions concerning gambling: preference for risk and time delay (the latter is called time preference in behavioral economics). While these two economic phenomena have often been treated separately, there is increasing evidence in favor of an interactive relationship [e.g., (*56*)]. Our computational model of anticipation explicitly offers an interaction between risk (prediction error) and delay (anticipation) because the former can enhance the value of the latter. This interaction creates well-documented effects, such as nonlinear coding of probabilities of anticipated rewards (*57*). It would be interesting to test our model’s predictions as to how pharmacological manipulations (e.g., on dopamine) affect risk and time (delay) preference, where dopamine is likely to be heavily involved in computing aRPE. Further studies may allow us to design a behavioral task for psychiatric interventions, in which patients can lessen their preference for addictive substances, or even their risk preference in general, because our model can find the optimal task parameters for each individual to achieve this goal.

We found that the hippocampus was involved in value computation arising from reward anticipation, through its coupling with the VTA/SN and the vmPFC. Both hippocampus-VTA/SN and hippocampus-(v)mPFC couplings have been extensively reported previously in animal studies as well as in some human studies [e.g., (*16*, *17*, *20*, *45*)]. In rodents, hippocampus-PFC coupling has been shown to be gated by neurons in the VTA (*16*). Oscillatory synchronization has also been reported in the PFC-VTA-hippocampus axis in rodents performing a working memory task. Our finding is consistent with a previous observation in humans showing that activity in VTA influences the baseline activity in posterior hippocampus (*45*). The posterior hippocampus, which we report in our PPI analysis, has also been linked to future simulation that we think likely relates to our model’s anticipation utility computation. Our functional connectivity analyses suggest that an aRPE signal encoded in the VTA/SN affects a functional coupling between the hippocampus and the vmPFC, which encode the enhancement of the utility of anticipation; this can be tested in future studies involving pharmacological manipulations (e.g., on dopamine). Because the hippocampus has a rich anatomical structure, further studies will illuminate how different parts of the hippocampus contribute to value computation arising from reward anticipation.

Neuroeconomic studies show that people make decisions between goods in different categories, by expressing the value of those goods in a so-called common currency primarily encoded in the vmPFC. Here, we found that the utility of anticipation is expressed in the vmPFC [please also see (*7*)]. This invites an alternative interpretation of previously reported ramping activity in the vmPFC while waiting for rewards [e.g., (*58*)] in terms of an anticipation-sensitive value signal, which has been interpreted as a reward-timing signal.

An alternative interpretation of our behavioral results is that participants do not like uncertainty. However, a previous study using the same task with aversive outcomes has shown that people avoid advanced information when the outcomes are aversive (*49*), while another study has also shown that a preference for advanced information is valence dependent (*24*). These findings are consistent with our model’s predictions but contradict simple uncertainty avoidance. In our model, advanced information can boost negative anticipation for an aversive outcome [i.e., dread (*2*, *26*)], leading to an avoidance of (negative) advanced information. Further studies will illuminate how advanced information modulates dread in the brain, possibly through hippocampal coding of a sustained signal during a waiting period for no reward (fig. S14; note: people assigned negative value to no reward in our task, confirmed by our model fitting and self-reports), and may suggest that a similar circuit presented here is involved in this computation.

As is the case for value of a reward [whose psychological roots have been shown to be very complex (*29*)], the psychological roots of anticipation utility are likely to be complex. While we acknowledge that we had no control over what participants were thinking while waiting for outcomes in the scanner, participants’ informal self-reports were largely consistent with the idea that reward predictive cues made participants more excited while waiting for the reward. We acknowledge that other psychological interpretations of our computational model are possible, as is the case for the roots of reward in a standard reinforcement learning model (*29*). For example, we note an influential suggestion (*4*) that future uncertainty drives other forms of anticipatory utility, such as anxiety. We did not consider this notion directly in our computational model, but in our model, an agent can experience a mixture of positive and negative utilities of anticipation according to the probabilities of these outcomes (please see Materials and Methods). It would be interesting to study how this mixed anticipatory utility of our model relates to the notion of anxiety (*4*), which may help the design of more effective psychiatric interventions for anxiety disorders. Also, in this current study, we used a primary reward (image) instead of a secondary reward (money); it would be interesting to administer our task using a secondary reward. We used primary reward inspired by the classic study of the utility of anticipation (*1*); however, recent studies implicate that similar results will be obtained with monetary reward (*6*, *24*).

Last, our study offers an alternative view to a long-standing problem in neuroscience and machine learning. We refer here to the so-called temporal credit assignment problem, which raises the issue of how neurons operating on a time scale of milliseconds learn relationships on a behaviorally relevant timescale (such as actions and rewards in our task). Designing a machine learning algorithm that overcomes this problem remains a challenge. Cognitively, our computational model suggests that the anticipation of future reward could serve as an aid to solve this problem, because a sustained anticipation signal can bridge the temporal gap between a reward predictive cue and an actual reward. A recent physiological study demonstrated that synaptic plasticity in hippocampal pyramidal neurons (e.g., place cells) can learn associations on a behaviorally relevant time scale, with the aid of ramping-like, slow, external inputs in a realistic setting (*59*). This has been shown to arise out of a slow input that can trigger a slow ramp-like depolarization of synaptic potential, which, in turn, unblocks *N*-methyl-d-aspartate (NMDA) receptors, leading to synaptic learning that spans a duration of seconds (*59*). Thus, our results suggest that a slow anticipatory utility signal in the vmPFC that is sustained throughout long delay periods (or the sustained, coupled, activity in the hippocampus) could serve as such input to neurons in the hippocampus, bridging the temporal gap over behavioral time scales. A dopaminergic input from the VTA/SN to the hippocampus may facilitate this type of learning (*17*).

In summary, we identify a novel neural substrate for computing the utility value arising from anticipation. Our results implicate that a functional coupling of three distinctive brain regions links the arrival of advanced information to resolve future uncertainty to the boosted utility of anticipation. We suggest that this boosted anticipatory utility drives a range of behaviors, including information-seeking, addiction, and gambling. Our study may also provide seed for designing individually tailored interventions for psychiatric disorders.

## MATERIALS AND METHODS

### Participants

Thirty-nine self-declared heterosexual male participants (*21*) were recruited from the University College London (UCL) community. Participants provided informed consent for their participation in the study, which was approved by the UCL ethics committee.

### Experimental task

The task was a variant of that in (*8*), which itself was inspired by a series of animal experiments into information-seeking or observing behavior [e.g., (*22*, *25*)]. At the beginning of each trial, a pair of task-information stimuli (hourglass and partially covered human silhouette) were shown, along with two choice targets. The number on the hourglass indicated how long the participants had to wait until seeing a reward or no reward, where 1/2, 1, 2, 4, and 8 hourglass meant 1, 5, 10, 20, and 40 s of waiting time, respectively. The other stimulus, a partially covered human silhouette, indicated the probability of seeing a reward, specified by the area of the uncovered semicircle (5, 25, 50, 75, and 95% chance of rewards). Two lateral rectangular targets were presented as choices: the immediate-information target marked as “Find out now” and the no-information target marked as “Keep it secret.” The positions of the hourglass and the covered silhouette were kept the same every trial, but the locations of choice targets were randomly alternated between left and right on each trial.

The participants were required to choose between left and right targets by pressing a button within 3 s. Once the participants chose a target, one of the three cues appeared in the center of the screen. If the participants chose the immediate-information target, then a cue that signaled upcoming reward or no reward appeared on the screen until the onset of reward or no reward. If the participant chose the no-information target, then a cue that signaled no information about reward appeared on the screen. The meaning of the cues was fully instructed to participants beforehand. The meanings of the cues were counterbalanced across participants. To ensure immediate consumption, rewards were images of attractive female models from a set that had previously been validated as being suitably appetitive to heterosexual male participants (*8*, *21*); reward images were presented for 1 s. Images were chosen randomly from the top 100 highest-rated pictures that were introduced in (*21*). No image was presented more than twice to the same participants. In case of no reward, an image signaling absence of a reward was presented for 1 s. In either case, a blank screen was presented for 1 s before starting a new trial. These timings were set to reduce the timing uncertainty, which may cause prediction error that can interfere with our model’s value computation.

Participants were fully instructed about the task structure, including the meaning of stimuli about the probability and delay conditions, as well as the advanced information cues. Then, participants underwent extensive training that consisted of three tasks: a variable-delay but fixed-probability task, a fixed-delay but variable-probability task, and a variable-delay and variable-probability task. This ensured that participants had fully learned the task and had adequately developed preferences before being scanned. Scanning was split into three separate runs, each of which consisted of 25 trials that covered all conditions once. Trial orders were randomized across participants. Participants had a break of approximately 30 s between runs.

### Computational model

We used the model described in (*8*). Briefly, following Loewenstein’s suggestion that the anticipation of rewards itself has hedonic value (*1*, *2*) (e.g., participants enjoy thinking about rewards while waiting for them), we extended a standard reinforcement learning framework to include explicit reward anticipation, which is often referred to as savoring (*1*). The model’s innovation is to suggest that the utility of anticipation can be boosted by RPEs associated with advanced information about upcoming rewards (*8*). We note that savoring here is a mathematically defined economics term and is different from (although may be related to) savoring in positive psychology (the acts of enhancing positive emotions).

To describe the model formally, consider a task in which if a participant chooses the immediate-information target, then they receive at *t* = 0 a reward predictive cue *S*^+^ with a probability of *q*, or a no-reward predictive cue *S*^−^ with a probability of 1 − *q*. Subsequently, the subject receives a reward or no reward at *t* = *T*( = *T*_delay_), with a value of *R*^+^ or *R*^−^, respectively. In our recent experiment, we found that participants assigned a negative value to an absence of reward (*8*), but this is not necessary to account for preference for advanced information that has been observed in animals (*3*, *25*).

On the basis of the observation that participants prefer to delay consumption of certain types of rewards, Loewenstein proposed that participants extract utility while waiting for reward (*1*, *2*, *26*). Formally, the anticipation of a future reward *R*^+^ at time *t* is worth *a*(*t*) = *R*^+^*e*^−ν^+^(*T* − *t*)^, where ν^+^ governs its rate. Including *R* itself, and taking temporal discounting into account, the total value of the reward predictive cue, *Q*_*S*^+^_, is

$$\begin{array}{cc} Q_{S^{+}} & =\eta \;V^{\left[\text{anticipation}\right]}+V^{\left[\text{reward}\right]}\\ & =\eta \;\int_{0}^{T}e^{-\gamma^{+}t\prime }a(t\prime )\mathrm{dt}\prime +R^{+}e^{-\gamma^{+}T}\\ & =\eta \frac{R^{+}}{\nu^{+}-\gamma^{+}}(e^{-\gamma^{+}T}-e^{-\nu^{+}T})+R^{+}e^{-\gamma^{+}T} \end{array}$$ (1)

where η is the relative weight of anticipation, γ^+^ is the discounting rate, and *T* is the duration of delay until the reward is delivered. In a prior work, η had been treated as a constant that relates to subjects’ ability to imagine future outcomes (*1*); however, we proposed that it can vary. The size of modulation is determined by the δ_aRPE_ at the time of the predicting cue (*8*). Our proposal was inspired by findings of the dramatically enhanced excitement that follows such cues (*25*). A simple form of boosting arises from the relationship

$$\eta =\eta_{0}+C|\delta_{\text{aRPE}}|$$ (2)

where η_0_ specifies the base anticipation and *C* determines the gain. That anticipation is boosted by the absolute value of aRPE is important in applying our model to comparatively unpleasant outcomes (*8*). The boosting is sustained throughout a waiting period.

The total value of the no-reward predictive cue, *Q*_*S*^−^_, is then

$$\begin{array}{cc} Q_{S^{-}} & =\eta \;\int_{0}^{T}e^{-\gamma^{-}t\prime }a(t\prime )\mathrm{dt}\prime +R^{-}e^{-\gamma^{-}T}\\ & =\eta \frac{R^{-}}{\nu^{-}-\gamma^{-}}(e^{-\gamma^{-}T}-e^{-\nu^{-}T})+R^{-}e^{-\gamma^{-}T} \end{array}$$ (3)

Following our previous work, we assumed that γ = γ^+^ = γ^−^.

An aRPE affects the total cue values *Q*_*S*^+^_ and *Q*_*S*^−^_, which, in turn, affect subsequent aRPEs. Therefore, the linear ansatz for the boosting of anticipation by aRPE (Eq. 2) could lead to instability due to unbounded boosting. This instability could account for maladaptive behavior such as addiction and gambling. However, in a wide range of parameters, this ansatz has a stable, self-consistent, solution. In our experiment, the aRPE for the reward and no-reward predictive cues can be expressed as

$$\delta_{\text{aRPE}}^{S^{+}}=Q_{S^{+}}-(qQ_{S^{+}}+(1-q)Q_{S^{-}})$$ (4)

$$\delta_{\text{aRPE}}^{S^{-}}=Q_{S^{-}}-(qQ_{S^{+}}+(1-q)Q_{S^{-}})$$ (5)

which are, assuming the linear ansatz

$$\left\{ \begin{array}{c} \delta_{\text{aRPE}}^{S^{+}}=(1-q)((\eta_{0}+C|\delta_{\text{aRPE}}^{S^{+}}|)A^{+}+B^{+}\\ -((\eta_{0}+C|\delta_{\text{aRPE}}^{S^{-}}|)A^{-}+B^{-})\\ \delta_{\text{aRPE}}^{S^{-}}=-q((\eta_{0}+C|\delta_{\text{aRPE}}^{S^{+}}|)A^{+}+B^{+}-\\ \left((\eta_{0}+C|\delta_{\text{aRPE}}^{S^{-}}|)A^{-}+B^{-}\right) \end{array}\right.$$ (6)

where

$$\left\{ \begin{array}{c} A^{+}=\frac{R^{+}}{\nu^{+}-\gamma }(e^{-\gamma T}-e^{-\nu^{+}T})\\ A^{-}=\frac{R^{-}}{\nu^{-}-\gamma }(e^{-\gamma T}-e^{-\nu^{-}T})\\ B^{+}=R^{+}e^{-\gamma T}\\ B^{-}=R^{-}e^{-\gamma T} \end{array}\right.$$ (7)

Assuming that *R*^−^ ≤ 0 and 0 ≤ *R*^+^, Eq. 6 implies that $\delta_{\text{aRPE}}^{S^{+}}>0$ and $\delta_{\text{aRPE}}^{S^{-}}<0$. With this, Eq. 6 can be reduced to

$$\left\{ \begin{array}{c} \delta_{\text{aRPE}}^{S^{+}}=\frac{\left(1-q)(\eta_{0}(A^{+}-A^{-})+B^{+}-B^{-}\right)}{1-C((1-q)A^{+}-qA^{-})}\\ \delta_{\text{aRPE}}^{S^{-}}=\frac{-q(\eta_{0}(A^{+}-A^{-})+B^{+}-B^{-})}{1-C((1-q)A^{+}-qA^{-})} \end{array}\right.$$ (8)

Because (η_0_(*A*^+^ − *A*^−^) + *B*^+^ − *B*^−^) > 0, in order that $\delta_{\text{aRPE}}^{S^{+}}>0$ and $\delta_{\text{aRPE}}^{S^{-}}<0$ hold for all *q* and *T*, the denominators must be positive for all 0 ≤ *q* ≤ 1 and 0 ≤ *T*. In other words

$$1-C((1-q)A^{+}-qA^{-})>0$$ (9)

for 0 ≤ *q* ≤ 1 and 0 ≤ *T*, or $C<\frac{1}{\left((1-q)A^{+}-qA^{-}\right)}$, for 0 ≤ *q* ≤ 1 and 0 ≤ *T*. This means that $C<\frac{1}{\text{max}(A^{+},|A^{-}|)}$ for 0 ≤ *T*. It is straightforward to show that *A*^+^ takes its maximum at $T=\frac{\text{ln}(\frac{\gamma }{\nu^{+}})}{\gamma -\nu^{+}}$, and ∣*A*^−^∣ at $T=\frac{\text{ln}(\frac{\gamma }{\nu^{-}})}{\gamma -\nu^{-}}$. Thus, the condition that the linear ansatz gives a stable self-consistent solution is

$$C<\text{min}(\frac{\gamma }{R^{+}}{\left(\frac{\gamma }{\nu^{+}}\right)}^{\frac{\nu^{+}}{\gamma -\nu^{+}}},\frac{-\gamma }{R^{-}}{\left(\frac{\gamma }{\nu^{-}}\right)}^{\frac{\nu^{-}}{\gamma -\nu^{-}}})$$ (10)

In our model fitting, we imposed this stability condition. Violating it could account for maladaptive behavior such as addiction and pathological risk-seeking. We generated choice probability from our model by taking a difference between the expected value of immediate information target and that of no-information target and taking it through sigmoid with a noise parameter σ (*8*).

An alternative to imposing such a stability condition would be to assume that boosting saturates in a nonlinear manner (*8*)

$$\eta =\eta_{0}+c_{1}\text{tanh}\;(c_{2}|\delta_{\text{aRPE}}|)$$

However, the model’s qualitative behavior does not depend strongly on the details of the aRPE dependence of anticipation (*8*). Hence, we only used the linear ansatz in our analysis in the current study.

For our model comparison, we also fit a model with no anticipation η = 0 and a model with anticipation but that is not boosted by aRPE, i.e., *C* = 0.

### Model’s fMRI predictions (parametric and time-varying regressors)

Our computational model makes specific predictions about temporal dynamics of anticipatory, reward, value signals during waiting periods, and unique aRPE signals at predictive cue onsets. Using the parameters (MAP estimates) for each participant, we generated the following variables for each participant as parametric regressors for the fMRI analysis.

The temporal dynamics of anticipatory utility signal for positive domain at time *t* during waiting period until reward onsets *t* = *T* are

$$V_{\text{Ant}.,+}(t)=\frac{R^{+}(\eta_{0}+C|\delta_{\mathrm{pe}}^{\left[S^{+},q,T\right]}|)}{\nu^{+}-\gamma }(e^{-\gamma (T-t)}-e^{-\nu^{+}(T-t)})$$ (11)

For the negative domain, they are

$$V_{\text{Ant}.,-}(t)=\frac{R^{-}(\eta_{0}+C|\delta_{\mathrm{pe}}^{\left[S^{-},q,T\right]}|)}{\nu^{-}-\gamma }(e^{-\gamma (T-t)}-e^{-\nu^{-}(T-t)})$$ (12)

We expressed these as two separate regressors. When the outcome was uncertain, i.e., after receiving a no-information cue, but would be given with a probability *q* (or 1 − *q*), the anticipatory utility values (Eqs. 11 and 12) were multiplied with *q* (or 1 − *q*).

Because aRPEs explicitly enter the value function of the immediate information via boosting, aRPE and the value of the immediate information target that influence each other needed to be computed in a self-consistent manner (Eq. 5). We assumed that the consistency was achieved for participants through their extensive training sessions. The aRPE $\delta_{\mathrm{pe}}^{\left[+/-,q,T\right]}$ are determined for each delay *T* and reward probability *q* condition self-consistently (see below). After a no-information choice, these signals are scaled by the probability of reward *q* or no reward 1 − *q* (and no prediction errors). Note that we set *R*^+^ = 1 without loss of generality.

The discounted reward signal at *t* during the waiting period is expressed as

$$V_{\text{Reward},+}(t)=R^{+}e^{-\gamma (T-t)}$$ (13)

while the discounted no-reward signal at *t* is

$$V_{\text{Reward},-}(t)=R^{-}e^{-\gamma (T-t)}$$ (14)

Note that the anticipation utility signal is an integral of (discounted) anticipation urgency signal

$$V_{\text{Ant}.\text{Urgency},+}(t)=R^{+}(\eta_{0}+C|\delta_{\mathrm{pe}}^{\left[S^{+},q,T\right]}|)e^{-\nu^{+}(T-t)}$$ (15)

and

$$V_{\text{Ant}.\text{Urgency},-}(t)=R^{-}(\eta_{0}+C|\delta_{\mathrm{pe}}^{\left[S^{-},q,T\right]}|)e^{-\nu^{-}(T-t)}$$ (16)

which we also included to the GLM.

The aRPE at information cue onsets are computed for each condition (*q*, *T*) self-consistently according to Eq. 8. That is

$$\left\{ \begin{array}{c} \delta_{\mathrm{pe}}^{\left[S^{+},q,T\right]}=\frac{\left(1-q)(\eta_{0}(A^{+}-A^{-})+B^{+}-B^{-}\right)}{1-C((1-q)A^{+}-qA^{-})}\\ \delta_{\mathrm{pe}}^{\left[S^{-},q,T\right]}=\frac{-q(\eta_{0}(A^{+}-A^{-})+B^{+}-B^{-})}{1-C((1-q)A^{+}-qA^{-})} \end{array}\right.$$ (17)

where *A*^+/−^ and *B*^+/−^ are given by Eq. 7. In our analysis, we put positive and negative aRPE as a single parametric regressor at information cue onsets. Because the aRPE is expressed as the difference between the model’s presented cue value and the model’s expected cue value in Eq. 5, we also tested a region that is positively correlated with the model’s presented cue value and negatively correlated with the model’s expected cue value in Eq. 5.

Note that the aRPE signal is different from other conventional prediction error signals, including the so-called SPEs (*43*)

$$\left\{ \begin{array}{c} \delta_{\mathrm{spe}}^{S^{+}}=|1-q|\\ \delta_{\mathrm{spe}}^{S^{-}}=|0-q| \end{array}\right.$$ (18)

and a standard RPE signal with reward value alone (we can obtain this by setting *C* = η_0_ = 0 in Eq. 17)

$$\left\{ \begin{array}{c} \delta_{\mathrm{pe}-\text{standard}}^{\left[S^{+},q,T\right]}=(1-q)(B^{+}-B^{-})\\ \delta_{\mathrm{pe}-\text{standard}}^{\left[S^{-},q,T\right]}=-q(B^{+}-B^{-}) \end{array}\right.$$ (19)

which we used for a confirmatory analysis.

### Behavioral model fitting

We used a hierarchical Bayesian, random effects analysis (*8*). In this, the (suitably transformed) parameters **h***_i_* of participant *i* are treated as a random sample from a Gaussian distribution with means and variance **θ** = {**μ**_θ_, **Σ**_θ_} characterizing the whole population of participants, and we find the maximum likelihood values of **θ**.

The prior distribution **θ** can be set as the maximum likelihood estimate

$$\begin{array}{c} \theta^{\mathrm{ML}}\approx {\text{argmax}}_{\theta }\{p(D|\theta )\}\\ ={\text{argmax}}_{\theta }\{\prod_{i=1}^{N}\int dh_{i}\;p(D_{i}|h_{i})p(h_{i}|\theta )\} \end{array}$$ (20)

We optimized **θ** using an approximate expectation-maximization procedure. For the *E* step of the *k*th iteration, a Laplace approximation gives us

$$m_{i}^{k}\approx \text{argma}x_{h}\{p(D_{i}|h)p(h|\theta^{k-1})\}$$ (21)

$$p(h_{i}^{k}|D_{i})\approx N(m_{i}^{k},\Sigma_{i}^{k})$$ (22)

where $N(m_{i}^{k},\Sigma_{i}^{k})$ is a normal distribution with mean $m_{i}^{k}$ and covariance $\Sigma_{i}^{k}$ that is obtained from the inverse Hessian around $m_{i}^{k}$. For the *M* step

$$\mu_{\theta }^{k+1}=\frac{1}{N}\sum_{i=1}^{N}m_{i}^{k}$$ (23)

$$\Sigma_{\theta }^{k+1}=\frac{1}{N}\sum_{i=1}^{N}(m_{i}^{k}m_{i}^{kT}+\Sigma_{i}^{k})-\mu_{\theta }^{k+1}\mu_{\theta }^{k+1T}$$ (24)

For simplicity, we assumed that the covariance $\Sigma_{\theta }^{k}$ had zero off-diagonal terms, assuming that the effects were independent.

### Model comparison

We compared the goodness of fit for different computational models according to their iBIC scores (*8*). Briefly, in this method, we sampled parameters randomly from the estimated distributions and tested how these randomly sampled models can predict the individual subject’s choice. We analyzed log-likelihood of data *D* given a model *M*, log *p*(*D*∣*M*)

log p(D|M)=∫d θp(D|θ)p(θ|M) (25)

$$\approx -\frac{1}{2}\text{iBIC}=\text{log}\;p(D|\theta^{\mathrm{ML}})-\frac{1}{2}|M|\text{log}\;|D|$$ (26)

where iBIC is the integrated Bayesian information criterion, ∣*M*∣ is the number of fitted prior parameters, and ∣*D*∣ is the number of data points (total number of choice made by all subjects). Here, log *p*(*D*∣θ*^ML^*) can be computed by integrating out individual parameters

$$\text{log}\;p(D|\theta^{\mathrm{ML}})=\sum_{i}\text{log}\int dhp(D_{i}|h)p(h|\theta^{\mathrm{ML}})$$ (27)

$$\approx \sum_{i}\text{log}\;\frac{1}{K}\sum_{j=1}^{K}p(D_{i}|h^{j})$$ (28)

where we approximated the integral as the average over *K* samples **h***^j^*’s generated from the prior *p*(**h**∣θ*^ML^*).

### fMRI data acquisition

We acquired MRI data using a Siemens Trio 3-T scanner with a 32-channel head coil. The Echo planar imaging (EPI) sequence was optimized for minimal signal dropout in striatal, medial prefrontal, and brainstem regions: 48 slices with 3-mm isotropic voxels with a repetition time of 3.36 s, an echo time of 30 ms, and a slice tilt of 30°. In addition, field maps (3-mm isotropic, whole brain) were acquired to correct the EPIs for field-strength inhomogeneity.

### fMRI data preprocessing

We used SPM12 (Wellcome Trust Centre for Neuroimaging, UCL, London) for standard preprocessing and image analysis. The standard preprocessing includes the following: slice-timing correction; realigned and unwarped with the field maps that were obtained before the task; coregistration of structural T1-weighted images to the sixth functional image of each subject; segmenting structural images into gray matter, white matter, and cerebrospinal fluid; normalizing structural and functional images spatially to the MNI space; and spatially smoothing with a Gaussian kernel with full width at half maximum of 8 mm. The motion correction parameters were estimated from the realignment procedure and were included to the first-level GLM analysis.

### fMRI GLM analysis

We performed a standard GLM analysis with SPM, with high-pass filter at 128 s. We regressed fMRI time series with GLMs that consist of onset regressors (the presentations of the initial screen, the presentations of cues, and the presentation of outcomes), our model’s signals that we described in Materials and Methods (parametric regressors: model’s aRPE at cues and reward or no reward at outcome; model’s time-varying regressors: anticipatory utility signals for positive and negative outcomes, expected value signals for positive and negative outcomes, anticipatory urgency signals for positive and negative outcomes), and nuisance regressors. The onsets of cues preceding the shortest delay (1 s) was separately modeled so that the prediction errors at the cues were not affected by reward. The model’s predictive signals were generated for each of the anticipatory periods, using the model that was fit to each participant, which were then convolved with the canonical HRF function. We added nuisance parameters that consist of movement estimated from preprocessing, large derivatives of movement between volumes that were larger than 1 mm, boxcar function during the anticipatory periods, and boxcar function for each experimental run. In our confirmatory analysis, we also added boxcar function during the anticipatory periods that was parametrically modulated by constant expectation of reward, parametrically modulated cue presentation with SPEs. Please see Model’s fMRI predictions (parametric and time-varying regressors) for the full equations.

When we illustrated the time course of BOLD signals, we used the data that were high-pass–filtered (128 s), and nuisance regressors were also regressed out.

### Fourier phase-randomization test

A recent study has reported potentially widespread false-positive correlations between slowly varying signals in neuroscience, such as value signals in the ventral striatum (*33*). To address this potential concern, we introduced a Fourier phase-randomization test. In this, we first transformed our model’s predicted signal (e.g., the utility of anticipation signal which continuously changes over time) into the Fourier space. Then, we randomized the phase of each frequency without disturbing the power. Randomization was performed by taking a random value from the uniform distribution between −π and π for each frequency component. It is also possible to permute the phase across frequency components. The signal was then transformed back to the original space. By replacing the original (prerandomized) regressor in the GLM by this phase-randomizing regressor, we ran the standard GLM analysis. This procedure was repeated for each participant over 100 times. We then performed a second-level analysis for the randomized regressor, where the group was constructed by selecting one GLM result randomly from each participant across all participants. To correct family-wise error, we selected the maximum correlation score across the whole family of voxels (e.g., the whole brain) from this second-level analysis, instead of the voxels that show the peak in our original analysis. We repeated this second-level analysis 1000 times to create a null distribution, consisting of the maximum correlation value in the family of voxels. This allows us to ask whether the observed correlation score is significantly great across the family of voxels (in our case, the whole brain). Therefore, our test is corrected for the family-wise error.

Another possibility is to take an average value in a given cluster and use it as a statistic. Please see figs. S8 and S9.

### Regions of interests

The anatomical masks for the ROIs for the VTA/SN and the hippocampus were taken from (*39*) and (*46*), respectively. The anatomical ROIs for control analyses were taken as follows: LC from (*60*), amygdala from automated anatomical labeling (AAL) atlas (amygdala-L + amygdala-L-aal) and vmPFC from AAL atlas (Frontal-Inf-Orb-L + Frontal-Inf-Orb-R + Rectus-L + Rectus-R). Functional masks were used for illustrative purposes in our confirmatory analyses, which are described in the main text.

### PPI analysis

We performed PPI analysis with a single GLM, which contained (i) the BOLD signal of VTA/SN, (ii) a PPI regressor that is an interaction between the BOLD signal of VTA/SN and model’s anticipation utility signal, (iii) the BOLD signal of vmPFC, and (iv) a PPI regressor that is an interaction between the BOLD signal of vmPFC and the model’s aRPE signal, as well as other onset/movement regressors that we included in our original analysis.

The vmPFC’s seed activity was defined as the first eigenvalue of the BOLD signal in the cluster that correlated with our model’s anticipation utility (*P* < 0.05, whole-brain FWE-corrected), and VTA/SN’s seed activity was defined as the eigenvalue of the BOLD signal in the cluster that was significantly correlated with our model’s aRPE at cues [*P* < 0.05, FWE small volume–corrected (*39*)]. Note that both aRPE and anticipation utility signals were controlled in our PPI analysis.

## Supplementary Material

aba3828_SM.pdf

## SUPPLEMENTARY MATERIALS

Supplementary material for this article is available at http://advances.sciencemag.org/cgi/content/full/6/25/eaba3828/DC1

## Appendix

View/request a protocol for this paper from *Bio-protocol*.
