# Supplementary material for: The value of what’s to come: Neural mechanisms coupling prediction error and the utility of anticipation
Source: Sci Adv. 2020 Jun 19;6(25):eaba3828. doi: 10.1126/sciadv.aba3828 (PMC7304967; doi:10.1126/sciadv.aba3828)
Supplement: aba3828_SM.pdf [file aba3828_SM.pdf]

Science Advances
AAAS

advances.sciencemag.org/cgi/content/full/6/25/eaba3828/DC1

Supplementary Materials for

# The value of what's to come: Neural mechanisms coupling prediction error and the utility of anticipation

Kiyohito Iigaya*, Tobias U. Hauser, Zeb Kurth-Nelson, John P. O'Doherty, Peter Dayan, Raymond J. Dolan

*Corresponding author. Email: kiigaya@caltech.edu

Published 19 June 2020, *Sci. Adv.* **6**, eaba3828 (2020)
DOI: 10.1126/sciadv.aba3828

**This PDF file includes:**

Figs. S1 to S17
Table S1

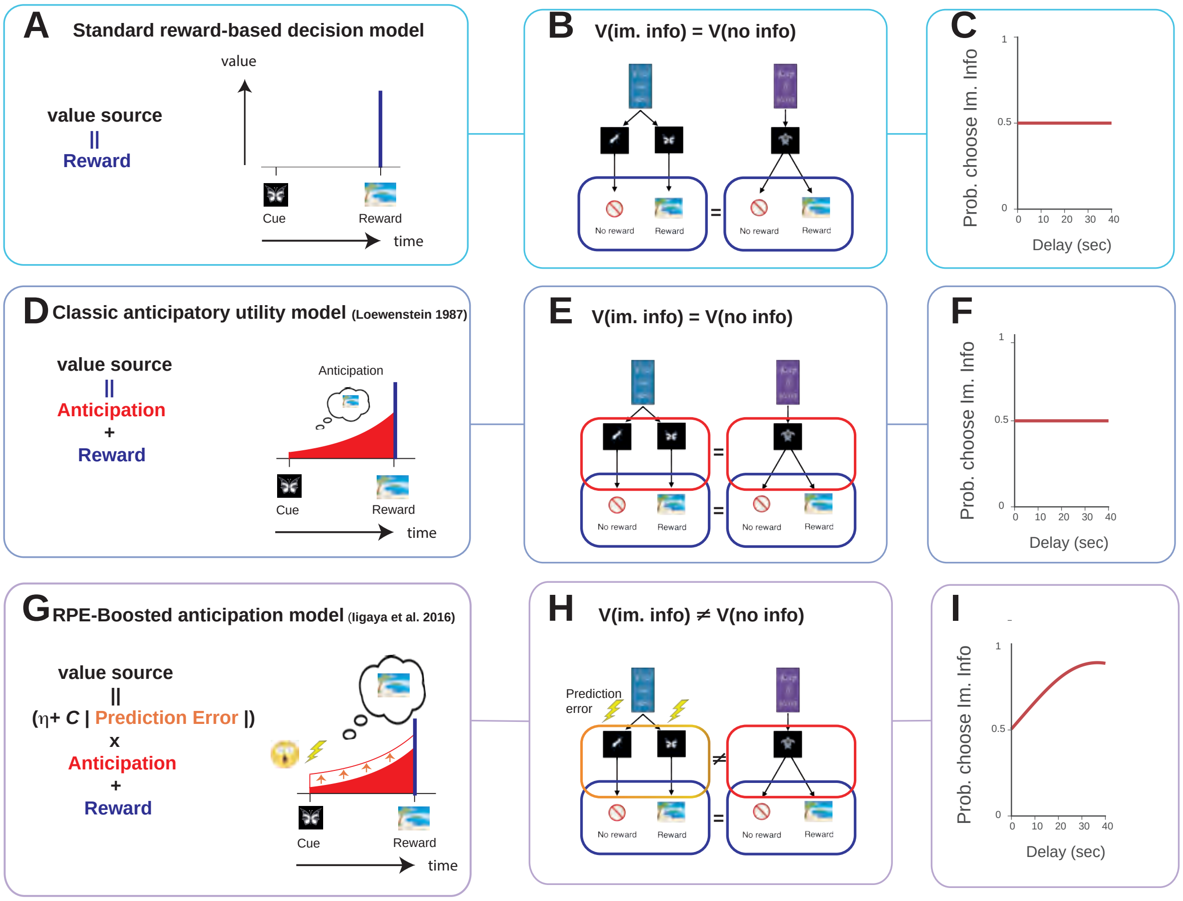


Figure S1: The mechanism by which the model predicts increasing preference for advanced information, as a function of the duration of the waiting period. (**A,B,C**). A classical reward-based decision-making model. This class of models assumes that a choice is made according to the attractive value of discounted future reward (A). Because there is no difference in the probability of obtaining reward between the Immediate-info-target and the No-Info-target, the model assigns the same value to the two choice targets (B). As a result, the model predicts no preference between the two targets across different delay conditions (C). (**D,E,F**). The classical behavioral economic model of the utility of anticipation.[1] The model assumes that people experience value from the anticipation of future reward, in addition to from consumption of the reward itself. Although this model can capture the well-documented behavior that subjects delay reward consumption, it assigns the same values to the Immediate-info-target and No-info-target in our task (E). As a result, this model also predicts indifferent choice between the two targets (F). (**G,H,I**). aRPE-boosting anticipation model.[8] Inspired by an observation of a dramatic increase in excitement after receiving the information that resolves uncertainty about upcoming reward,[25] this model hypothesizes that the utility of anticipation can be boosted by prediction errors associated with the reward predictive cues (G). Consequently, the value of the Immediate-info target can become greater than the value of the No-info target, as the duration of the wait becomes longer (H). As a result, the model predicts that subjects show stronger preference of the Immediate-info-target in longer delay conditions. This model has been previously validated in a series of behavioral experiments[8] (I).

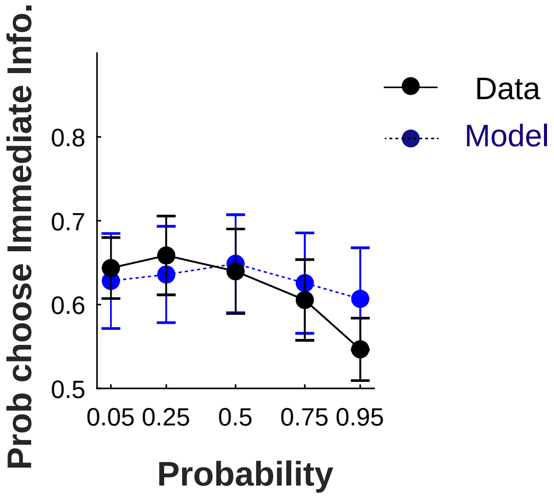


Figure S2: Our model (blue) captured choice preference over reward probability conditions in data (black). The error bars indicate the SEM of participants (n=39).

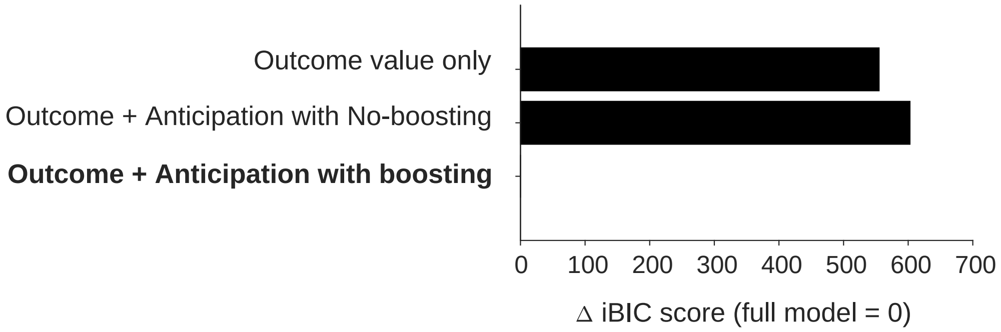


Figure S3: Our model comparison using integrated Bayesian Information Criterion (iBIC) strongly favors our full model with values of outcomes and aRPE-boosted anticipation, over a model with outcome values but no anticipatory utility, as well as a model with values of outcomes and anticipation that is not boosted by aRPE. All models included temporal discounting. A smaller score indicates a better model. The score is shown on the log-scale. Please see the Methods sections for the precise definitions of the models.

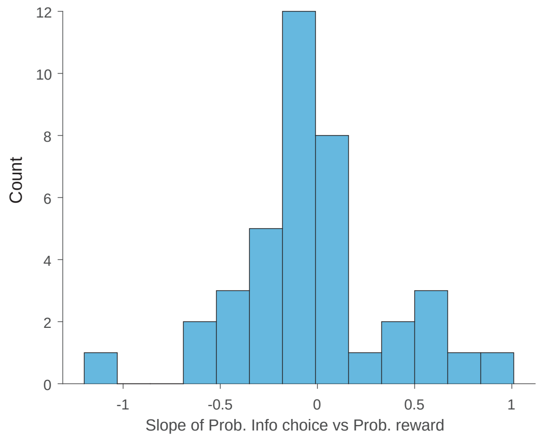


Figure S4: Heterogeneity amongst the participants in how their preferences for Info-target depended on the reward probability. The plot shows a histogram across the subjects of the slope of a linear fit to the probability of choosing the Info-target versus the probability of reward.

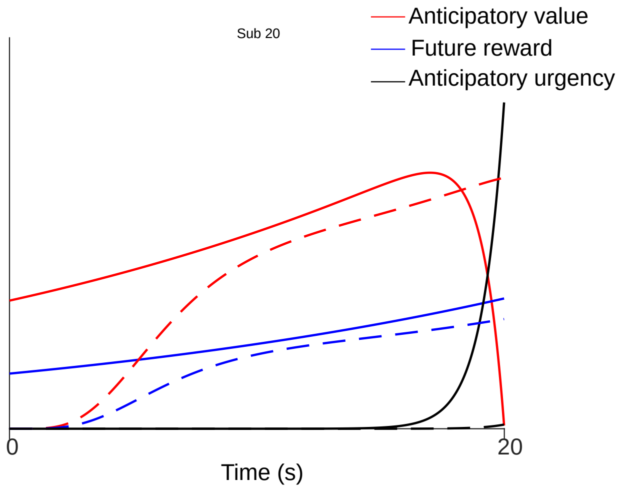


Figure S5: Prediction of fMRI signal in one subject (subjust 20). The anticipation utility signal (red). The discounted reward signal (blue). The anticipation stream signal (black). The dashed curves indicate HRF convoluted predictions for fMRI.

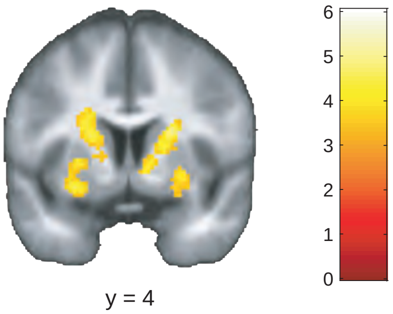


Figure S6: Voxels in dorsal caudate correlated with anticipation utility signal. The effects in caudate survived our phase-randomization test ($p < 0.001$ whole-brain FWE correction). The effects in posterior putamen did not survived the whole-brain correction. Voxels at $p < 0.001$ (uncorrected) are shown for display purposes.

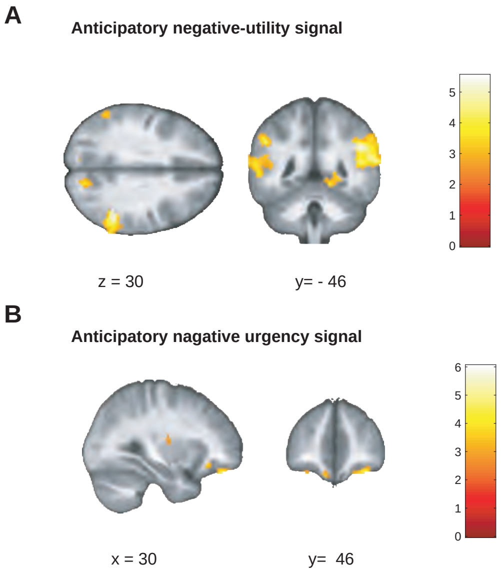


Figure S7: fMRI correlates in negative (no-reward) domain. (**A**) Correlation with anticipation utility signal for no-reward, which is in the negative domain. (**B**) Correlation with anticipatory urgency signal in the negative domain. In all panels, voxels at $p < 0.005$ uncorrected are shown for display purposes.

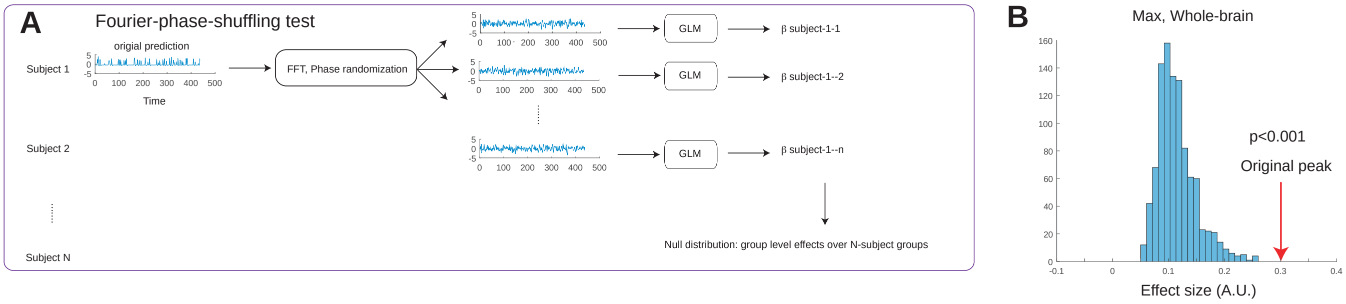


Figure S8: Our control analysis using phase-randomization validates the correlation between the model's anticipation utility signal and the fMRI signal. (**A**) Schematics of the analysis. In response to recent article about false-positive correlations between slow signals in neuroscience,[33] we performed a new analysis using phase-randomization of signals. For this, we first transformed our model's predicted anticipatory signal into the Fourier space. Then we randomized the phase of each frequency without disturbing the power, before transforming back to the original space. We then ran the standard GLM analysis using this regressor as a model's prediction to estimate the regression coefficient. We repeated this for each participant over 100 times (3,900 GLMs in total). We then randomly selected GLM results over participants (one from each participant) to perform a standard second-level analysis. We repeated this second-level analysis for 1,000 times to create a null distribution of the effect. The null distribution was constructed by taking the maximum correlation over each GLM result, and this was compared against the original. (**B**) Our test shows that our original correlation is significantly greater than by chance, compared to the null distribution constructed by the phase-randomization method ($p < 0.001$)

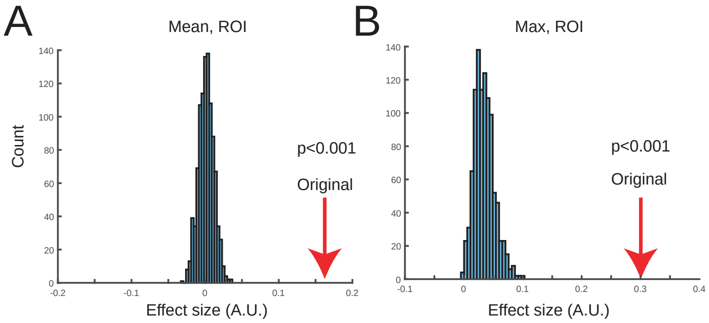


Figure S9: The same test as in Figure S8 but now performed in the vmPFC ROI is shown for an illustrative purpose. (**A**) The test is performed using the mean value of the vmPFC ROI. (**B**) The test is performed using the maximum value in the vmPFC ROI in each phase-shuffled GLM. Note that the null distribution is shifted toward zero compared to Figure S8B

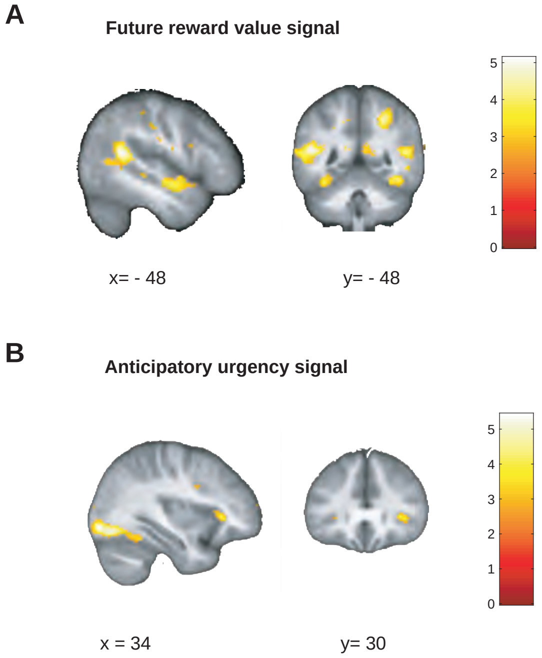


Figure S10: Correlates of our computational model's signals. (**A**) Correlations with discounted future reward signal. Regions in superior temporal gyrus survives the whole-brain FWE correction $p < 0.05$. (**B**) Correlations with anticipatory urgency signal. In all panels, voxels at $p > 0.005$ are shown for display purposes.

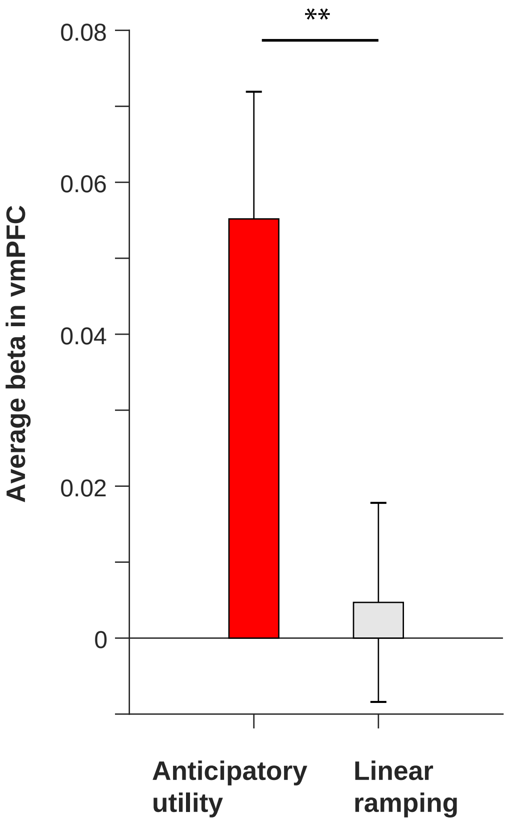


Figure S11: The average beta coefficients of the utility of reward anticipation at the vmPFC is significantly greater than the linear ramping signal (both included in the same GLM). The significance was tested by a permutation test, where two stars indicate $p < 0.01$.

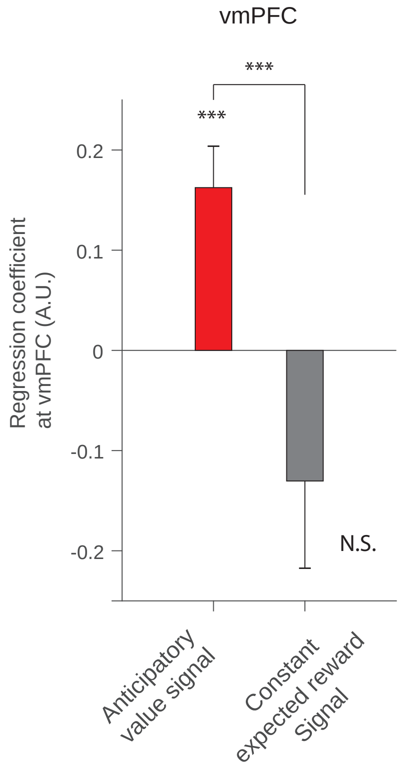


Figure S12: A confirmatory analysis shows that the vmPFC is more strongly correlated with our model's anticipatory utility signal than a standard expected reward value signal, defined by a boxcar regressor modulated by the probability of reward. In the GLM analysis with both regressors, average regression weights in the vmPFC cluster for the anticipation utility signal was significantly greater than the coefficients to the expected value signal ($p < 0.001$, permutation test). The average regression weights in the vmPFC cluster were significantly larger than zero for our model's predicted signal ($p < 0.001$ t-test, $t_{38} = 3.93$), but not significantly different from zero for the expected value signal. The error bars indicate the mean and SEM. Note that this is a confirmatory analysis.

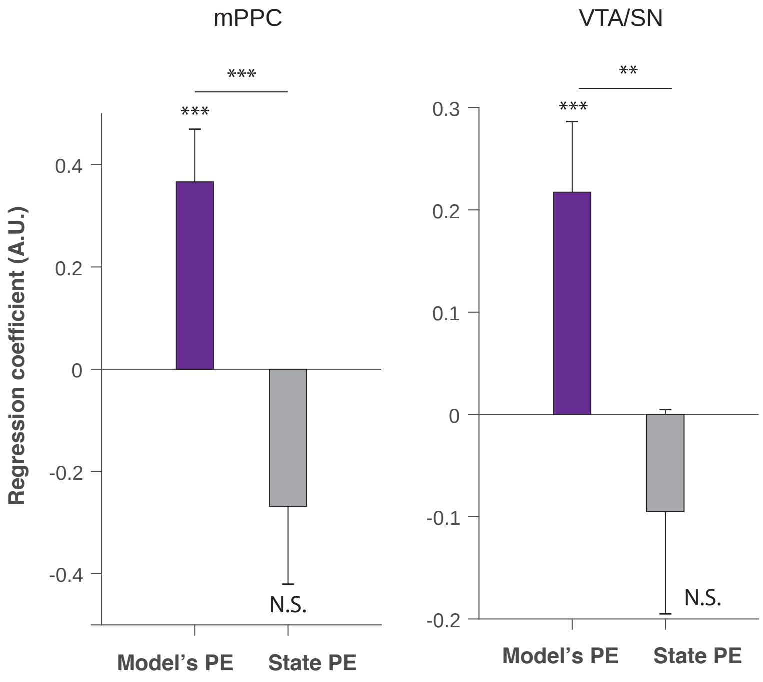


Figure S13: Our illustrative, confirmatory, analysis shows that BOLD signals in both the mPPC and the VTA/SN positively correlated with the model's anticipation-reward prediction error signal (aRPE), but not with a simpler, so-called state prediction error signal ($1 - p_{\text{reward}}$ when reward predictive cue was presented, $|0 - p_{\text{reward}}|$ when no-reward predictive cue was presented). The two regressors are included to the same GLM analysis. The differences between the average regression coefficients in the mPPC and in the VTA/SN were significant in the mPPC ($p < 0.001$, a standard permutation test in which we permuted the average regression coefficients), and the VTA/SN ($p < 0.001$ permutation test). The average correlation with the model's aRPE signal was significant both in the mPPC and the VTA/SN ($p < 0.001$ for the mPPC and the VTA/SN; t-test $t_{38} = 3.56$ and $t_{38} = 3.15$). The three stars indicate $p < 0.001$, and two stars indicate $p < 0.01$.

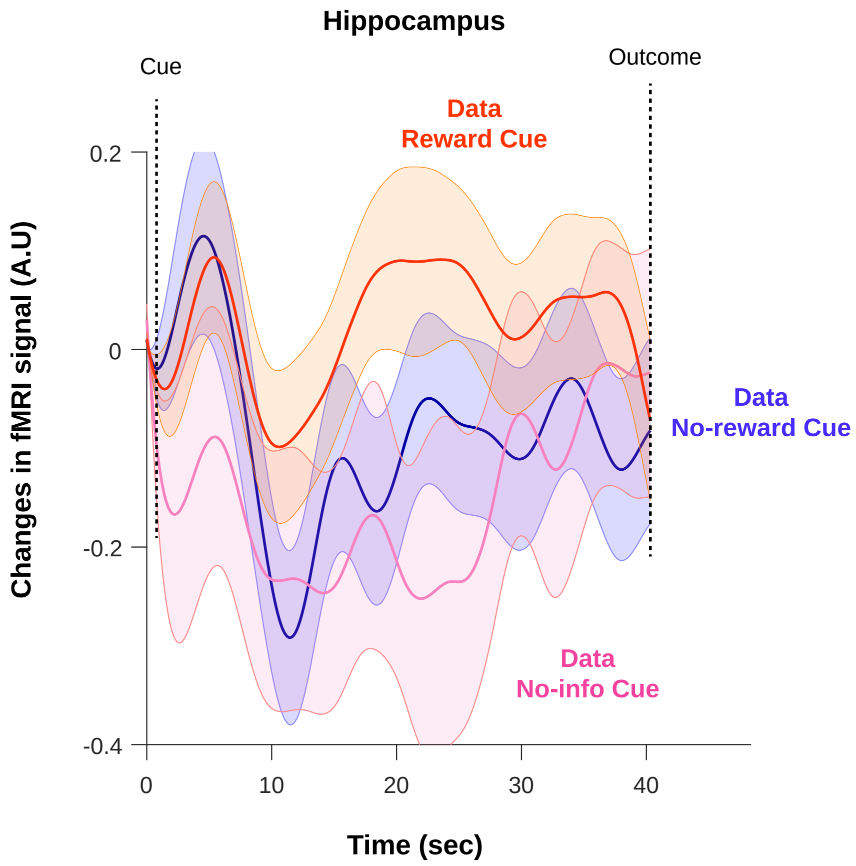


Figure S14: The temporal dynamics of the fMRI signal in hippocampus during anticipatory periods. Changes in activity averaged over participants after receiving a reward predictive cue (orange), after receiving a no-information cue (magenta), and after receiving a no-reward predictive cue (blue) are shown. The error bar indicates the SEM.

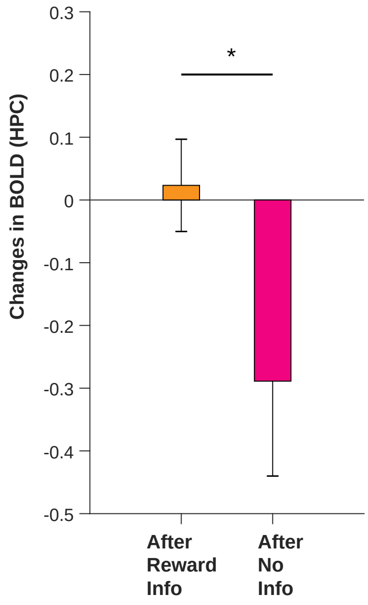


Figure S15: The average changes in BOLD in hippocampus after receiving a reward predictive cue or a no-information cue. The average change in BOLD signal since the cue presentation was calculated for each participant on each 40 sec delay condition. The first 10 sec of each delay period was excluded from the analysis. The star indicates a significance ($p < 0.05$), tested by a permutation test. For each trial, the BOLD at the cue presentation was set to zero. The error bars indicate the mean and SEM.

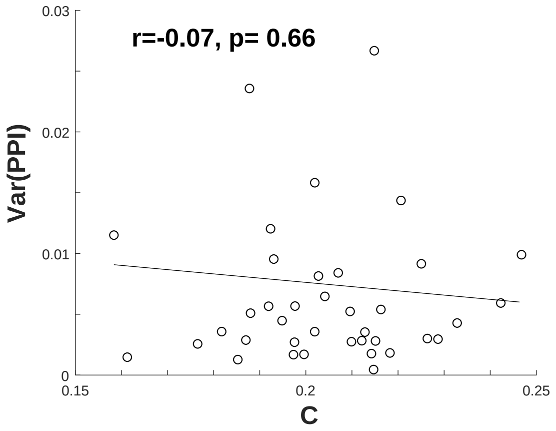


Figure S16: Correlation between the models parameter C and the PPI regressor that is used to estimate inter-subject correlation (Figure 5C). We found no correlation. We found no correlation ($p > 0.5$).

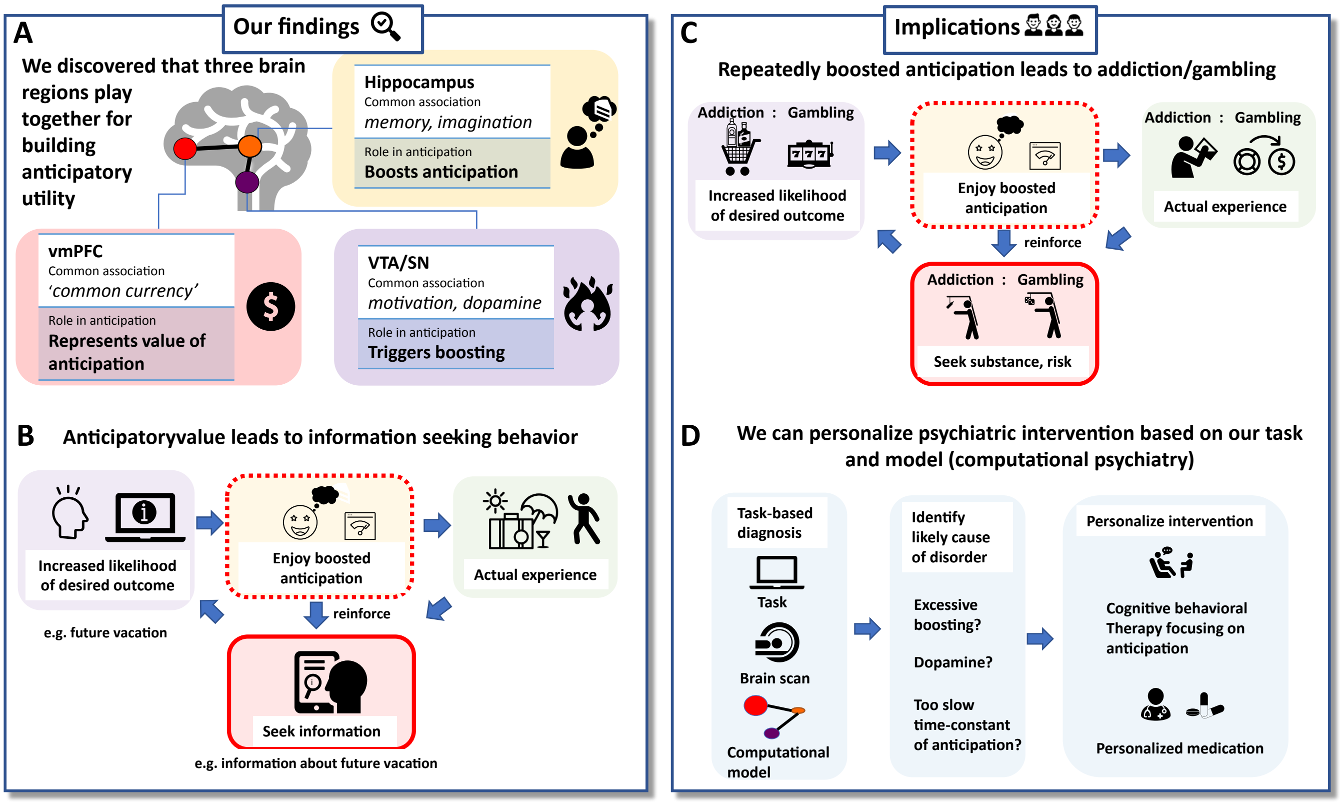


Figure S17: Summary of our findings and implications. (**A,B**). Our primary findings. (**A**). We uncovered three distinctive regions that compute anticipation utility and links it to advanced information in the brain. The vmPFC, often described as brain's common currency region, represented the utility of anticipation. The VTA/SN, regions associated with dopamine and motivation, triggers the boosting of anticipation when the likelihood of desired outcome increases. The hippocampus, a strong associate of memory and imagination, realized the boosting of anticipation. (**B**). We showed that anticipation utility can drive information-seeking behavior. Advanced information can increase the likelihood of a desired outcome, which in turn boosts the utility of anticipation. As a result, people can feel enhanced value from anticipation after receiving advanced information. Therefore people seek advanced information about their desired outcomes (information-seeking, or observing), as we confirmed in our current and past experiments.[8] (**C,D**). The implication of our study. (**C**). Over-boosted anticipation could lead to addiction and gambling. Purchasing alcohol or seeing '7-7-7' in a slot machine increases the likelihood of receiving desired outcome (e.g., drinking alcohol, receiving money from gambling). This can boost the anticipation utility. By repeating this many times, the subjective value of alcohol or gambling can also be boosted over and over, leading to pathological seeking for substance (addiction) and risk (gambling). Note that our computational model predicts that this over-boosting can happen only to individuals with a particular set of parameter values (e.g. strong boosting and weak discounting). (**D**). Our study can help to design personalized psychiatric interventions (computational psychiatry). Subjects perform a behavioral task in an MRI scanner and we fit our computational model to the behavior. We can identify likely causes of psychiatric disorders (e.g., addiction), by the subject's parameters estimated by our computational model and brain data. This will help design personalized psychiatric intervention, for example cognitive behavioral therapy focusing on aspects of anticipation, as well as medication targeted to specific neuromodulators (e.g., dopamine). Please see the Discussion section for further details and other implications of our study.

| parameters | $\gamma$ | $\nu^+$ | $\nu^-$ | $R^-$ | C | T | $\eta$ |
|---|---|---|---|---|---|---|---|
| mean | 0.03 ($sec^{-1}$) | 1.23 ($sec^{-1}$) | 1.29 ($sec^{-1}$) | -1.00 | 0.20 | 0.0079 | 2.41 |
| std | 0.025 ($sec^{-1}$) | 0.13 ($sec^{-1}$) | 0.084 ($sec^{-1}$) | 0.12 | 0.19 | 0.005 | 0.26 |

Table S1. The mean and the standard deviation of MAP estimates. The parameters were transformed to the model's native space.
